# Efficacy and Safety of Makabuhay (Tinospora rumphii) 25% Cream Versus Hydrocortisone 1% Cream in the Management of Mosquito Bite Reactions: Randomized Double-Blind Controlled Trial

**DOI:** 10.2196/50380

**Published:** 2023-11-08

**Authors:** Julius Garcia Gatmaitan, Jolene Kristine Garcia Gatmaitan-Dumlao, Johannes Dayrit, Ma Teresita Gabriel

**Affiliations:** 1 Gatmaitan Medical and Skin Center Baliuag, Bulacan Philippines; 2 Skines Aesthetic and Laser Center Baliuag, Bulacan Philippines; 3 Notre Dame De Chartres Hospital Baguio Philippines; 4 Department of Medicine Baguio General Hospital Baguio City Philippines; 5 Department of Dermatology Research Institute for Tropical Medicine Alabang Muntinlupa City Philippines; 6 Department of Dermatology De La Salle Health Sciences Institute College of Medicine Cavite Philippines

**Keywords:** tinospora, hydrocortisone, mosquito bite reaction, randomized controlled trial

## Abstract

**Background:**

Most insect bite reactions resolve spontaneously, but the inflammation and pruritus induced have been shown to decrease the quality of life. Previous studies have shown the potential anti-inflammatory properties of *Tinospora rumphii*.

**Objective:**

The aim of the study is to assess the efficacy and safety of *T rumphii* 25% cream versus hydrocortisone 1% cream in the management of local cutaneous reactions caused by mosquito bites.

**Methods:**

This study was a parallel-group, double-blind, randomized, placebo-controlled trial with a 1-week duration in a span of 3 months (June 2019 to August 2019). Participants were exposed to sterile noninfectious mosquitoes (*Aedes aegypti*) for 5-10 minutes to elicit cutaneous lesions. *Tinospora* 25% cream or hydrocortisone 1% cream was applied twice daily throughout the 7-day study period. Of the 70 participants screened for this study, which was approved by an institutional review board (IRB 2019-07) at the Dermatology Outpatient Department of the Research Institute for Tropical Medicine, Alabang, Muntinlupa, Philippines, 58 participants in total met the inclusion criteria and were randomized to treatment (*Tinospora*: n=29) and active control (hydrocortisone: n=29) groups.

**Results:**

In total, 58 participants were randomized to receive *Tinospora* cream (n=29) or hydrocortisone cream (n=29). All participants completed the follow-up. There was a significant decrease in lesion size in both groups from the first 15 minutes to day 7 (*P*<.001). Comparing the lesion size in both groups, there was a statistically significant decrease in lesion size in the first hour (*P*=.003) and after 24 hours (*P*=.03). On day 1, 10% (n=29) of participants in the hydrocortisone group and 7% (n=29) in the *Tinospora* group experienced complete resolution. On day 3, all participants experienced complete resolution. No adverse effects were documented.

**Conclusions:**

*Tinospora* 25% cream is safe, effective, and comparable to hydrocortisone 1% cream as an anti-inflammatory agent for mosquito bite reactions based on the decrease in lesion size, the proportion of participants with complete resolution of wheals, and improvement in pruritus intensity score using a visual analog scale. Long-term safety studies are recommended.

**Trial Registration:**

Philippine Health Research Registry PHRR230716-005932; https://www.herdin.ph/index.php/registry?view=research&layout=details&cid=5932

## Introduction

Although mosquitoes are well-known vectors of infectious diseases such as malaria, encephalitis, West Nile infection, and the yellow, dengue, and Zika fevers [1], these insects more frequently cause innocuous reactions on the skin when they feed on their hosts [2]. These cutaneous reactions to mosquito bites are caused by an immunologic response to proteins in mosquito saliva, and many who are bitten develop an immune response to these proteins [3]. Pruritus is a sensation limited only to the skin, which causes the desire to scratch. Certain stimuli like insect bites may cause intense allergic reactions and the production of immediate inflammatory responses in humans. Mosquito saliva induces activation of mast cells causing them to degranulate and secrete mediators, such as histamine, neutral proteases, and proteoglycans, and some cytokines, such as tumor necrosis factor-α. Allergic reactions in response to mosquito bites might be urticarial, tuberculin, or eczematoid [4]. Activation of these mediators causes vasodilation and enhancement of vascular permeability and stimulatory sensory nerves and can manifest clinically as wheals, edema, and pruritus [5]. The immunological responses to mosquito saliva in humans can range from the more common immediate and delayed local cutaneous reactions to the rarer, more severe, generalized reactions such as urticaria or angioedema of the skin and mucous membranes [6,7]. Immediate cutaneous reactions to mosquito bites often present as wheals with surrounding erythema that peak at 20 minutes, while delayed cutaneous reactions manifest as pruritic, indurated papules that peak at 24 to 36 hours and resolve over the next 7 to 10 days [8]. Lymphocytes, immunoglobulin E (IgE), and immunoglobulin G contribute to the pathophysiology of local cutaneous reactions. Both serum mosquito salivary gland–specific IgE and immunoglobulin G levels correlate significantly with the size of the wheals seen in immediate local reaction to mosquito bites, while an increase in the number of lymphocytes correlates with delayed cutaneous reactions [3]. While most insect bite reactions resolve spontaneously within 7-10 days, the inflammation and pruritus caused by these bites are often bothersome and have even been shown to decrease the quality of life. A quality-of-life study performed by Halasa et al [9] explored the impact of mosquitoes in the community. Quality of life was measured using the EuroQOL EQ-5D descriptive system that explored the 5 dimensions mobility, self-care, usual activities, pain or discomfort, and anxiety or depression for each dimension that has 3 levels: no problems, some problems, and extreme problems. Of the 121 residents, the majority (54.6%) of the respondents considered mosquitoes to be a problem. In total, 59.5% of the residents said the presence of mosquitoes prevented them from enjoying their outdoor activities. Based on this study, mosquito bites during summer may be comparable to living with up to 2 risk factors for diabetes (ie, abdominal obesity, BMI of 28 or more, reported cholesterol problems, diagnosis of hypertension, or history of cardiovascular disease) or women experiencing menstrual disorders [9,10]. The incessant scratching of these pruritic lesions may also lead to secondary infections and lichenification that require further treatment [11]; hence, there is merit in stopping the itch-scratch cycle early in the history of the insect bite. There is no gold standard in the management of mosquito bite reactions. Topical corticosteroids, topical antipruritic agents (eg, calamine), and oral antihistamines are often prescribed for immediate and delayed mosquito bite reactions, but there is a lack of clinical evidence for the efficacy of these treatments and, in general, recommendations for these are based on expert opinion or clinical experience, and rarely, randomized controlled clinical trials [12]. Inflammation is defined as the local response of living mammalian tissues to injury due to any agent. It is a body defense reaction in order to eliminate or limit the spread of injurious agents. Depending upon the defense capacity of the host and duration of response, it is classified as acute or chronic. Among them, the main features of acute inflammation are the accumulation of fluid and plasma, intravascular activation of platelets, and recruitment of neutrophils as initial inflammatory cells. Histamine, 5-hydroxytryptamine, and bradykinin are the first detectable mediators in the early phase of carrageenan-induced inflammation, whereas prostaglandins are detectable in the late phase of inflammation [13].

*Tinospora rumphii*, synonymous with *Tinospora cordifolia* and *Tinospora crispa* and locally known as Makabuhay, is a large, glabrous, deciduous climbing shrub belonging to the family Menispermaceae, distributed in Southeast Asia [14]. In vitro and in vivo studies have demonstrated immunomodulatory [15], antiangiogenic [16], anti-inflammatory [17], and antipruritic [18] properties of *Tinospora* species. These properties have been postulated to be due to the alkaloids, diterpenoid lactones, glycosides, steroids, sesquiterpenoid, phenolics, aliphatic compounds, and polysaccharides present in the *T* *cordifolia* plant [19]. There are several studies on inflammation responding to *Tinospora* species. An in vitro study by Zalawadia et al [18] showed that *T cordifolia* showed an inhibitory effect on histamine-induced paw edema in mice. The mice were injected with 30 μg of histamine diphosphate to induce the paw edema and were subsequently given the test drugs (*Tinospora* extracts at increasing concentrations ranging from 125 to 1000 mg/kg) per orem. The negative control group was given saline, while the positive control group was given cetirizine, an antihistamine medication. *Tinospora cordifolia* treatment showed a significant reduction in paw volume as compared to the negative control group. Furthermore, as compared to the positive control group, *Tinospora* also demonstrated H1 antihistamine activity as well as mast cell stabilization properties. The authors of the study suggested that the antioxidant potential of *T cordifolia* may have reduced the reactive oxygen species present in the activation of the mast cells and subsequently prevented histamine release [19]. A literature search on clinical studies on the topical preparation of *Tinospora* yielded results for its antiscabies, antibiotic, and antiangiogenic properties [20]. Only an unpublished in vivo study by Delos Santos et al [20] showed that *T cordifolia* is stable in lotion form and is an effective anti-inflammatory and wound healing agent. Rat paw edema was induced using 0.1% mL of 1% formalin and was administered to the right hind paw of each rat subject. The application of 10% and 25% *T cordifolia* lotion and diclofenac gel (positive control) twice a day for 7 days showed a significant decrease in edema compared to the application of plain lotion base [20].

Local studies have demonstrated the safety of using *Tinospora* in both animals and humans. A study by Lagda and Galang [21] on patch testing of *T rumphii* cream on rabbits showed no irritation after 72 hours and the succeeding 7 days after the initial application. The cream can be used safely in concentrations of 25%, 50%, 75%, and 90% [21]. Galang et al [22] performed patch testing of the *T rumphii* cream on human participants, and their results also showed no irritation after 72 hours and the succeeding 2 weeks after the initial application and can be safely used in concentrations of 25%, 50%, and 90%.

To date, there are no published clinical trials investigating the use of *Tinospora species* on cutaneous reactions due to mosquito bites. Steroids have been previously used for mosquito bite reactions. Its misuse may be associated with adverse effects such as pigmentary changes, striae, skin atrophy, glaucoma (if used around the eyes), acneiform eruptions, and bruising, among others. The anti-inflammatory potential of *T rumphii* may provide a safe and natural alternative therapy for mosquito bite reactions. The general objective of our study is to compare the efficacy and safety of *T rumphii* 25% cream versus hydrocortisone 1% cream in the management of local cutaneous reactions caused by mosquito bites. Specifically, we will explore the proportion of participants experiencing complete resolution of wheals after 1 hour and on days 1, 3, and 7 of the study period; the lesion size after 1 hour and on days 1, 3, and 7 of the study period; the pruritus intensity scores using a visual analog scale (VAS) after 1 hour and on days 1, 3, and 7 of the study period; and the proportion of participants experiencing adverse effects.

## Methods

### Study Design or Study Area or Setting

This was a parallel-group, double-blind, randomized, placebo-controlled trial with a 1-week duration. This study was conducted at the Dermatology Outpatient Department and the Entomology Laboratory of the Research Institute for Tropical Medicine, Alabang, Muntinlupa, Philippines. The study was conducted over a span of 3 months from June 2019 to August 2019.

### Sample Size

The sample size was calculated using the formula for a difference in proportions, using a power of 80% and a 5% level of significance. The proportion of success for the *T cordifolia* group and the hydrocortisone group was assumed to be 75% and 45%, respectively, based on the results of a previous study [23,24]. Calculations indicated that a total of 38 participants were needed (19 participants in each study arm). We recruited 58 participants to allow for a 20% dropout rate.

α=level of significance=0.05, 1–β=power=0.80, *f*(α–β)=7.85

π_1_=the anticipated proportion in those in the control group=45%

π_2_=the anticipated proportion in those in the treatment group=75%



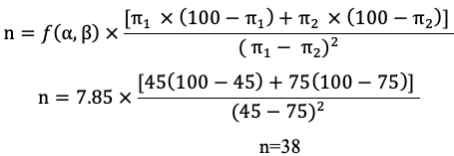



### Recruitment and Selection of Participants

Purposive sampling was used in this study. Participants were recruited through the following methods: (1) direct recruitment (talking with potential participants about the study and using considerable care that the person does not feel pressured to participate); (2) posting institutional review board (IRB)–approved advertisements on bulletin boards at the lobby, dermatology department, training center, and triage area; and (3) through referrals (physicians and colleagues).

### Study Population

The inclusion criteria are the following: healthy men or women aged between 18 and 60 years, participants willing to sign an informed consent form after having read and understood its content upon the investigator’s explanation, participants willing to comply with the study protocol requirements, and participants willing to have photos taken for documentation purposes.

Exclusion criteria are the following: participants with a history of severe reactions to mosquito bites (swelling and induration several centimeters in diameter, extensive periorbital swelling, lip swelling, and extensive limb swelling), pregnant or lactating women, participants with severe physical or mental illness, participants with active skin lesions or other dermatological disorders (ie, psoriasis, atopic dermatitis, and other eczemas), and participants with prior or current use of any oral antihistamines or corticosteroids in the past 2 weeks.

### Enrollment and Informed Consent

Those who fulfilled the inclusion criteria were recruited. Participants were brought to a private room, where the study protocol was explained. Written informed consent was secured by the investigators and participants. The participants were requested to return on a separate day for the study.

### Method of Randomization and Allocation Concealment

The study statistician generated a list of random numbers (simple randomization 1:1) using the computer-generated list of random numbers. A third party not directly involved in the study was assigned to the treatment arms as either 1 (hydrocortisone) or 2 (*Tinospora* species). Sealed opaque envelopes were used to allocate participants to either treatment (1 or 2). The envelope contained either 1 or 2, and assigned coinvestigators dispensed the jars accordingly. Only the pharmacist knew which code (1 or 2) corresponds to *Tinospora* cream or hydrocortisone cream. A diary card was given to participants and evaluated by investigators to ensure the compliance of participants. At the end of the study period, all case report forms (CRFs) were collected.

### Preparation of the Plant Material

The fresh mature stems of *T rumphii* were collected from the Sierra Madre mountain range at Infanta, Quezon Province, Philippines. These were authenticated by a botanist at the Bureau of Plant Industry (Multimedia Appendix 1 [25]). The samples were brought to the Department of Science and Technology—Industrial Technology Development Institute (DOST-ITDI) for preparation of the extracts. The organic constituents from the air-dried and chopped *T rumphii* stems were obtained by soaking the stems in 95% ethanol for 48 hours. The soaked solution was passed through Whatman No. 1 filter paper, and the filtrate was concentrated in a vacuum rotary evaporator at 60 °C in order to reduce the volume. The crude extracts yielded were submitted to DOST-ITDI for phytochemical analysis. A licensed pharmacist formulated the *Tinospora* 25% cream. Cultures of the cream were performed to ensure the absence of contaminants. The test product used for the active control group was hydrocortisone 1% cream (Marife C Biscocho Pharmacy). Both creams were unscented and similar in consistency. These were placed in identical white jars. The jars were coded (1 or 2) by the pharmacist. The investigators were unaware of the contents of each container applied to the participants.

### Study Intervention

**Figure 1:**
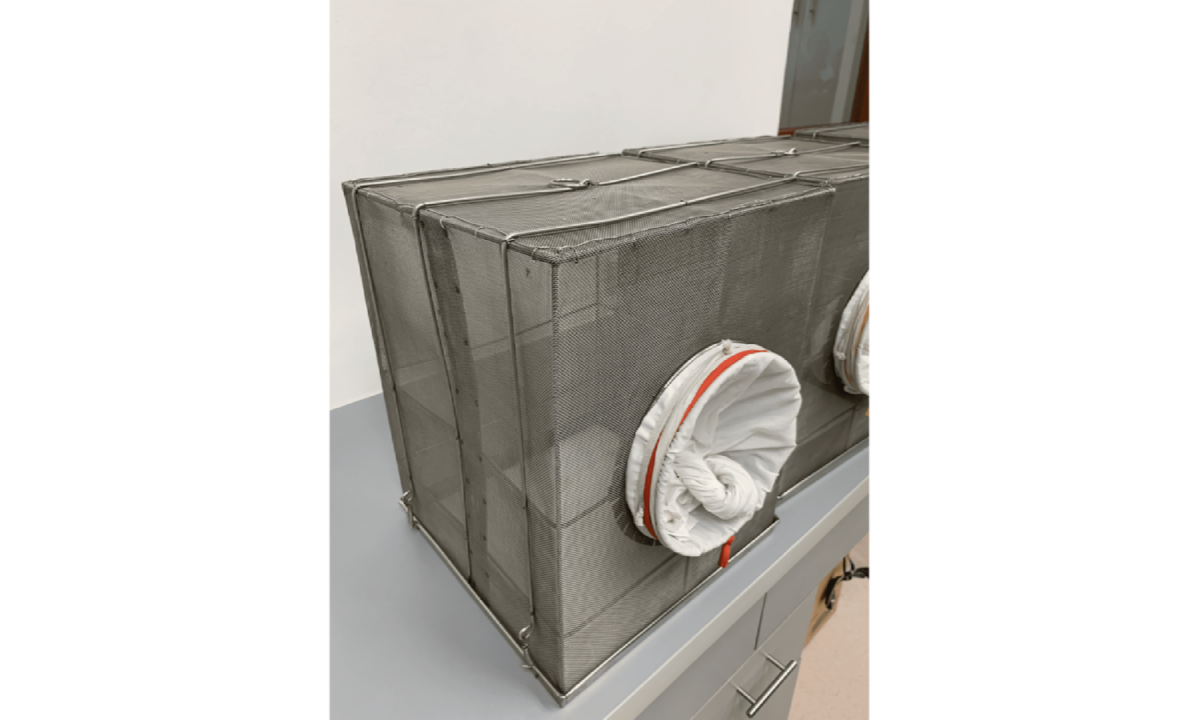
Cage containing female laboratory-reared *Aedes aegypti* mosquitoes.

A screened aluminum cage measuring 60×60×60 cm with a 15×15 cm diameter square opening fitted with cloth sleeves was used in this study (Figure 1). All the mosquitoes used were sterile and sucrose-starved for 48 hours prior to the test to ensure biting. These vectors have been grown in the laboratory under a controlled setup, are “clean” mosquitoes, and will not be able to transmit dengue or dengue hemorrhagic fever or other vector-borne diseases. Adequate safety precautions were ensured in order to prevent the introduction of “unclean” mosquitoes to the controlled setup. These measures are the use of a well-established entomology laboratory, aluminum cages with small holes that were kept closed, and a controlled opening of the aluminum cage for the arms of the participants.

**Figure 2:**
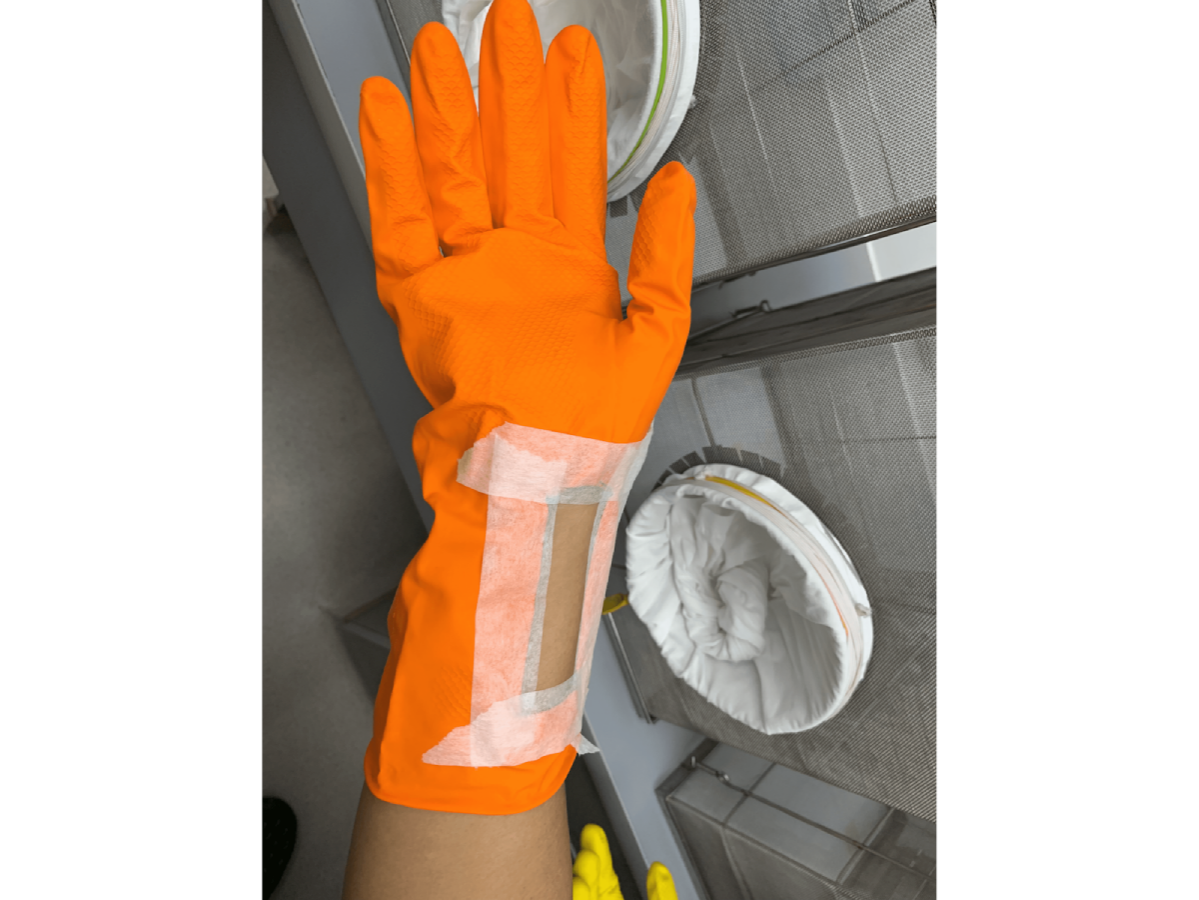
Rubber gloves with an 8 × 3.5 cm opening on the volar aspect.

The participants were instructed to wash their forearms with plain water and dry them with a clean towel prior to exposure to mosquitoes. The participants were asked to wear rubber gloves with an 8×3.5 cm opening on the volar aspect (Figure 2). This was done to limit the biting area.

**Figure 3:**
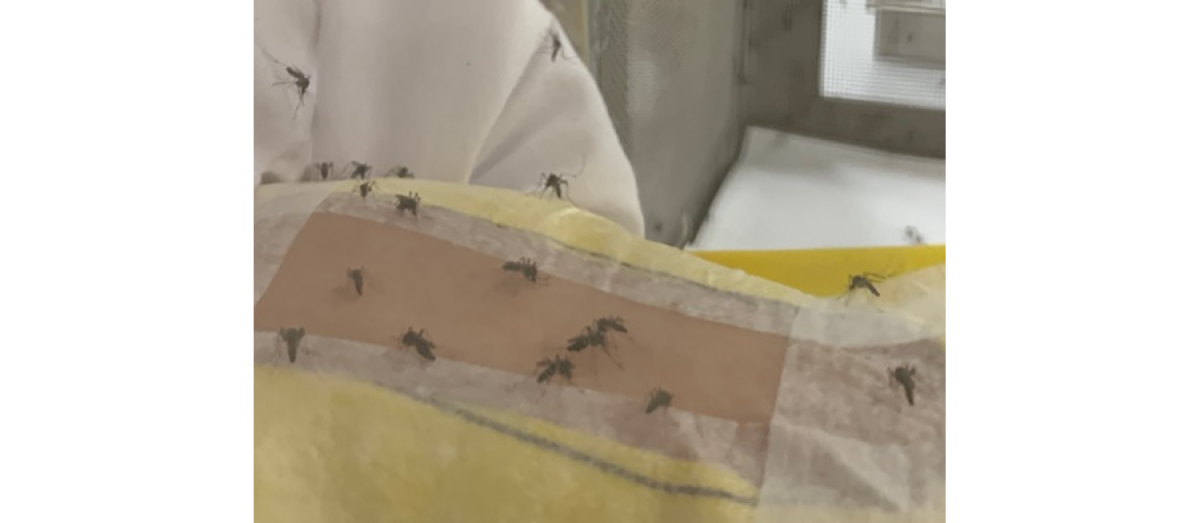
Mosquitoes feeding on the exposed volar aspect of the arm of participants.

Bite exposure with *Aedes aegypti* mosquitoes was performed between noon and 3 PM. A batch of 20 female laboratory-reared *A aegypti* mosquitoes were introduced in the cage every exposure period. Only 1 arm was exposed per participant. The range of exposure was 5-10 minutes, but once 5 mosquito bites were observed, the investigators asked the participant to remove the arm from the cage even before 5 minutes (Figure 3). The mosquitoes that have landed or fed were removed using a suction tube and were placed in a holding chamber and subsequently disposed.

**Figure 4:**
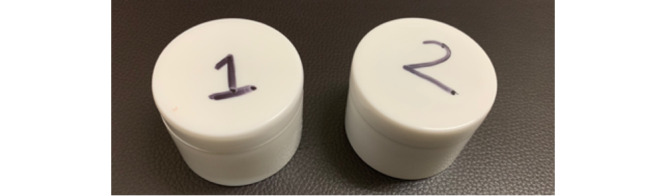
Jars containing either (1) hydrocortisone 1% cream and (2) *Tinospora* 25% cream.

During the whole test period, the participants were instructed not to rub or scratch their arms with mosquito bites. Any adverse cutaneous reaction such as burning and popular or vesicular or bullous eruptions was recorded. Each participant was given 1 jar of *Tinospora* 25% cream or hydrocortisone 1% cream (Figure 4). The first application of the cream was 15 minutes after the mosquito bites. The participants were advised to apply the cream twice a day thereafter on the lesions. To ensure compliance, they were provided a diary to record their application and were required to bring the test product container on every visit. The diary also contained an adverse reaction section to record any reactions. They were advised not to apply any other medication or emollient or take any oral antihistamines or corticosteroids during the study period. The same mild soap (Dove; Unilever) was given to all participants. Photos were taken after the exposure to the mosquitoes in the cage at 15 minutes (when there are mosquito bites), after 1 hour, and on days 1, 3, and 7 of the study period.

### Outcome Measures and Tools for Outcome Measurement

#### Primary Outcome Measure

Investigators assessed if there was no change, with worsening, with improvement, and complete resolution (0=no change, 1=with worsening, 2=with improvement, and 3=complete resolution). There must be no visible lesions seen on the volar aspect to be considered to have a complete resolution of lesions.

#### Secondary Outcome Measures

The lesion diameter was measured by the investigators using a single 150-mm stainless steel ruler (Robbins Instrument).

### Pruritus Score

**Figure 5:**
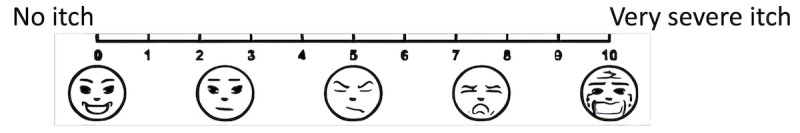
A 10 mm visual analog scale.

The VAS was used to evaluate the itching parameter. A VAS is an instrument that tries to measure a characteristic or attitude that is believed to range across a continuum of values and cannot easily be directly measured. Operationally, a VAS is usually a horizontal line, 10 mm in length, anchored by word descriptors at each end or with vertical lines and descriptors. The patient marks a line at the point that represents their perception of their current state. The VAS score is determined by measuring in millimeters from the left-hand end of the line to the point that the patient marks (Figure 5).

### Adverse Effect

Following the 4-point scale by the Standards of International Drug and Test Products, adverse cutaneous reactions were reported as shown in Table 1.

**Table 1:** Scoring of the adverse events using the 4-point scale (Standards of International Drug and Test Products).

Scale	Grade	Presentation
0	None	No adverse reactions
1	Mild	Erythema, itching, dryness, scaling, or stinging
2	Moderate	Burning, tenderness, or pain or the above mild
3	Severe	Vesicles, erosion, excoriation, or crusting or above mild or moderate

### Data Collection

All CRFs were filled up completely by the primary investigators. These were kept inside the cabinet at the research unit of the department. All forms were kept by the investigators and filed according to participant number. The initial wheal size 15 minutes after the mosquito bite was recorded. Participant assessment of change in lesion size, VAS, and pruritus score were recorded after 1 hour and on days 1, 3, and 7 of the study period. Adverse events (if any) were likewise recorded. Participants were required to bring their diary and product container to monitor compliance.

### Stopping Guidelines

This study was stopped in participants who experienced adverse reactions (severe pruritus, erythema, burning sensation, and vesicular or bullous eruption) to the creams or worsening of the mosquito bites (redness, warmth, swelling or induration that ranged from 2 to more than 10 cm in diameter, periorbital edema, extensive periorbital swelling, lip swelling, and extensive limb swelling). Participants were monitored based on World Health Organization guidelines for monitoring and reporting of adverse effects. Once a severe reaction occurred, the results were recorded, and participants were given the necessary medications. Unblinding was not necessary as the treatment would not have been different. Participants who did not comply with the protocol of applying test creams twice per day and those who applied other products or medications other than those stated in the protocol were considered to be withdrawn from the study. Dropouts referred to participants who were unable to return for assessment of lesions, and their outcomes were unknown by the end of the study period. The experiment was conducted in a controlled environment, with physicians trained in basic and advanced life support on standby. Although exceedingly rare, if participants experienced signs and symptoms of anaphylaxis, first aid was administered. Medications for anaphylaxis and other foreseeable adverse events, such as epinephrine, diphenhydramine, and intravenous and oral steroids, among others, were made accessible and available. Participants were brought to the nearby emergency room, which was within the vicinity of the outpatient clinic. Medical expenses for adverse events related to the study were shouldered by the study investigators. Participants could be withdrawn from the study due to (1) adverse events, (2) failure of therapy, (3) poor compliance, (4) lost to follow-up, and (5) voluntarily.

### Data Processing and Analysis

**Figure 6:**
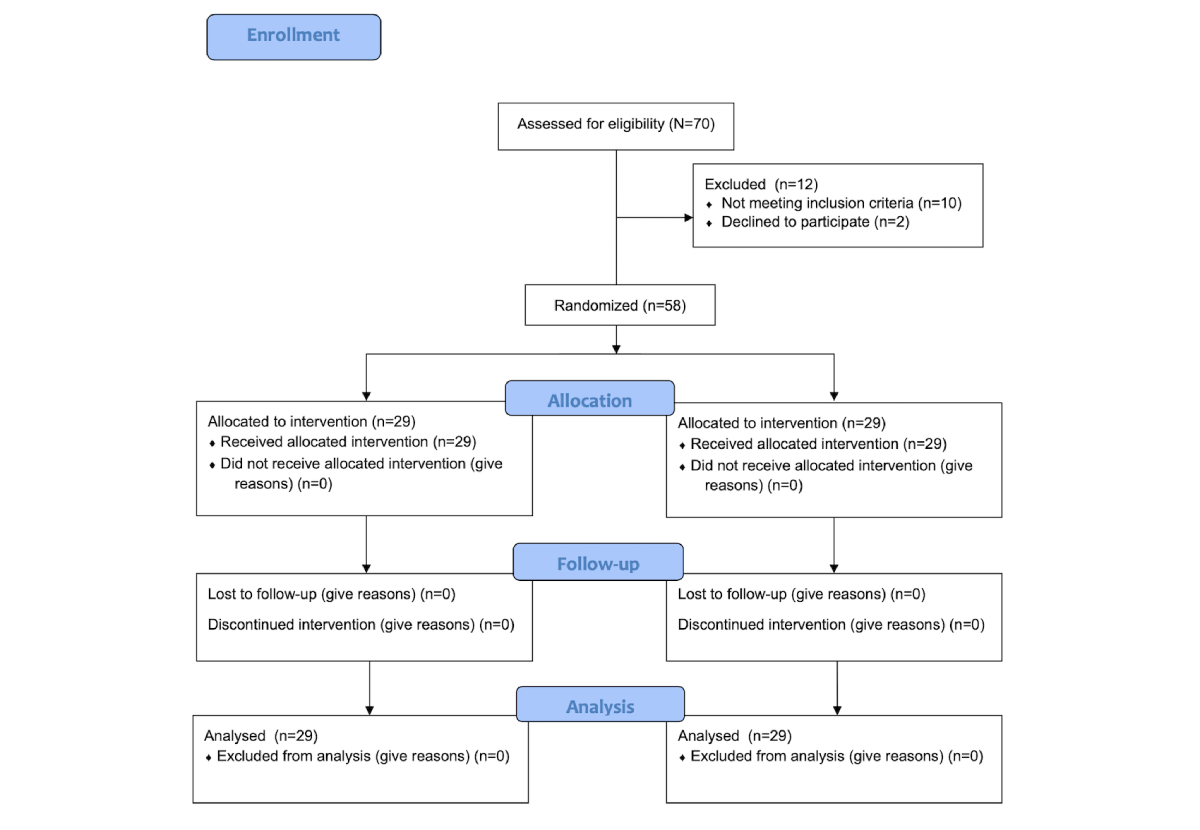
CONSORT 2010 flow diagram. Flow of participants through each stage of the trial (adapted from Schultz et al [26], with permission from Kenneth Schultz). CONSORT: Consolidated Standards of Reporting Trials.

To ensure blinding of the outcome assessor, a research assistant was hired for the collation of data. A standardized CRF approved by the IRB was used. CRFs were checked for completeness and errors by the primary investigators. Data were encoded by the research assistant in Microsoft Excel 2017 (Microsoft Corp). Collated data were given to the statistician for data analysis. All statistical analyses were performed using the R statistical package (version 3.2.0; R Foundation for Statistical Computing). Data on age were summarized using means and SDs. Frequencies and proportions were shown to describe sex and complete resolution of lesions. The size of lesions and pruritus scores were summarized using median and IQR. The *t* test for independent means was used to determine significant differences between the 2 groups in terms of age. It was a 2-tailed test. A chi-square test was conducted to determine if there were significant differences between the proportion of participants with complete resolution of mosquito bites (primary outcome measure) and the proportion of patients experiencing adverse effects. This test was also used to compare the age distribution of the 2 groups. The median of all lesion sizes and the median visual analog scores between hydrocortisone and *Tinospora* were compared using the Wilcoxon rank sum test. Friedman test was used to compare the change in lesion sizes and visual analog scores across a time period between hydrocortisone and *Tinospora*. No intention-to-treat analysis was done since there were no dropouts. Test results with *P*<.05 were considered to be statistically significant. The flow of participants through each stage of the trial is further seen in Figure 6 [26].

### Ethics Approval

This study was approved by the IRB of the Research Institute for Tropical Medicine (IRB 2019-07), registered at the Philippine Health Research Registry (PHRR230716-005932), and conducted in accordance with the tenets of the Declaration of Helsinki 2013 and National Ethical Guidelines for Health Research 2017.

## Results

### Phytochemical Analysis

In total, 500 mL of *Tinospora* extracts were sent to DOST-ITDI for qualitative phytochemical analysis. The results of the analysis are shown in Table 2. Sterols and triterpenes were strongly positive in the specimen submitted. Alkaloids, saponins, and tannins, as well as traces of glycosides, were found in the stem extracts of *Tinospora* that were used in our study.

**Table 2:** Qualitative analysis of phytochemicals present in the stem extract of Tinospora rumphii.

Sample code—sample—sample description/identification and test parameter^a^			Result^b^		Method used, Evans [27]
**ICS-2019-0466—extract—brownish turbid extract in a glass jar, marked as Makabuhay extract, approximately 500 mL**					
	Sterols	+++		Liebermann-Burchard test	
	Triterpenes	+++		Liebermann-Burchard test	
	Flavonoids	–		Magnesium turning test	
	Alkaloids	++		Mayer test	
	Saponins	++		Froth test	
	Glycosides	+		Fehling test	
	Tannins	++		Ferric chloride test	

^a^Units are inapplicable considering that the tests conducted are qualitative.

^b^Legend: (+) traces, (++) moderate, (+++) abundant, and (–) absence of constituent.

### Study Population

Of the 70 participants screened, 58 met the inclusion criteria and were randomized to treatment (*Tinospora*, n=29) and active control (hydrocortisone, n=29) groups (Figure 1). All patients completed the follow-up period. There were no dropouts and withdrawals in the study. Baseline characteristics of the study population are summarized in Table 3. The mean age of participants given hydrocortisone was 37 (SD 7) years, while participants given *Tinospora* were younger with a mean age of 31 (SD 5) years.

**Table 3:** Demographic profile of patients with mosquito bite treated with hydrocortisone and Tinospora rumphii.

Demographic profile	Hydrocortisone (n=29)	*Tinospora* (n=29)	*P* value
Male sex, n (%)	11 (38)	12 (41)	.79^a^
Female sex, n (%)	18 (62)	17 (59)	—^b^
Age (years), mean (SD)	37 (7)	31 (5)	.01^c^

^a^Significance set at *P*<.05 using the chi-square test.

^b^Not available.

^c^Significance set at *P*<.05 using the *t* test for independent means.

### Primary Outcome Measure

All participants treated with *T rumphii* 25% cream and hydrocortisone 1% cream showed improvement in lesion size after 1 hour. After 1 day, 10% (n=3) of participants given hydrocortisone had complete resolution compared to 7% (n=2) of those given *Tinospora* (*P*=.33), but this was not statistically significant. On the third day, 100% (n=29) of participants from both treatment groups had complete resolution of mosquito bites (Table 4).

**Table 4:** Proportion of participants with complete resolution of mosquito bites after treatment with hydrocortisone and Tinospora rumphii.

Time period			Hydrocortisone (n=29), n (%)		*Tinospora* (n=29), n (%)		*P* value^a^	
**After 1 hour**								N/A^b^
	No change	0 (0)		0 (0)				
	Worsened	0 (0)		0 (0)				
	Improved	29 (100)		29 (100)				
	Total clearing	0 (0)		0 (0)				
**After 1 day**								.33
	No change	0 (0)		2 (6.9)				
	Worsened	0 (0)		0 (0)				
	Improved	26 (90)		25 (86)				
	Total clearing	3 (10)		2 (7)				
**After 3 days**								N/A
	No change	0 (0)		0 (0)				
	Worsened	0 (0)		0 (0)				
	Improved	0 (0)		0 (0)				
	Total clearing	29 (100)		29 (100)				

^a^Significance set at *P*<.05 using the chi-square test.

^b^N/A: not applicable.

### Secondary Outcome Measures

**Figure 7:**
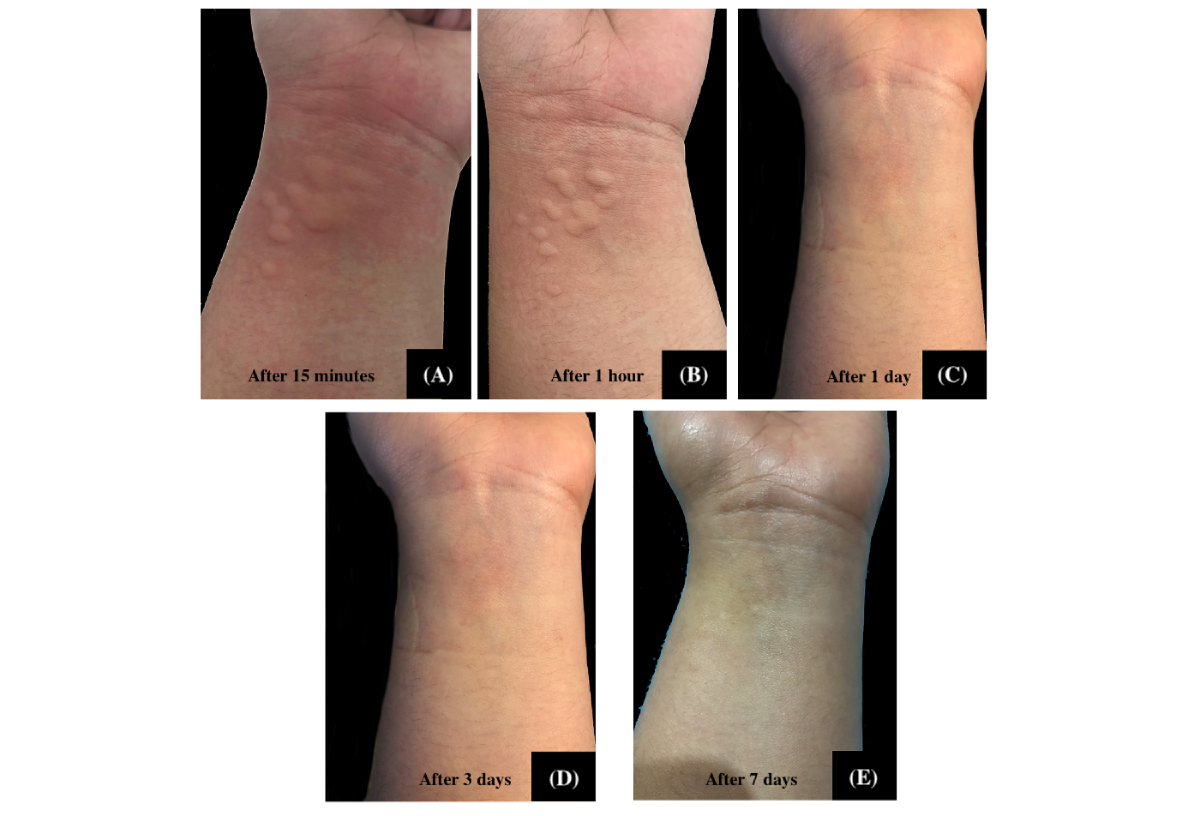
Representative photos showing the effect on lesion sizes treated with *Tinospora* 25% cream.

**Figure 8:**
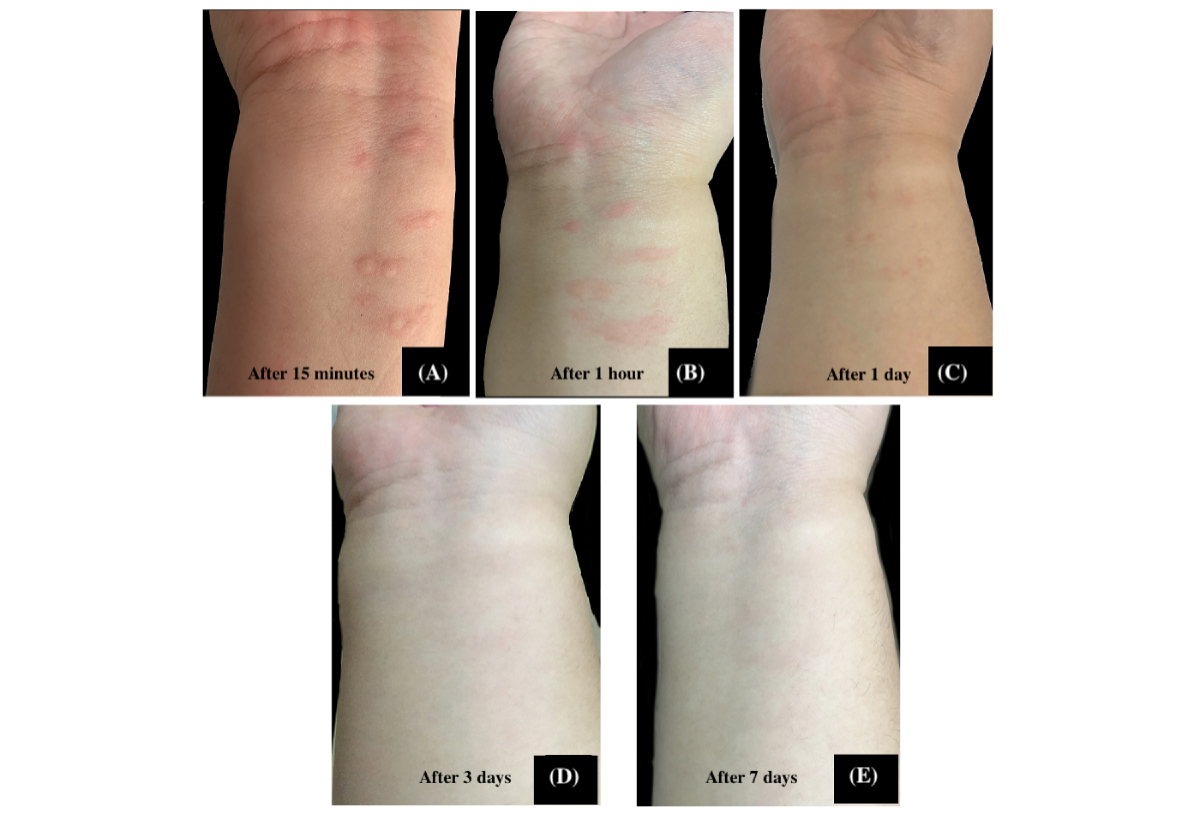
Representative photos showing the effect on lesion sizes treated hydrocortisone 1% cream.

**Figure 9:**
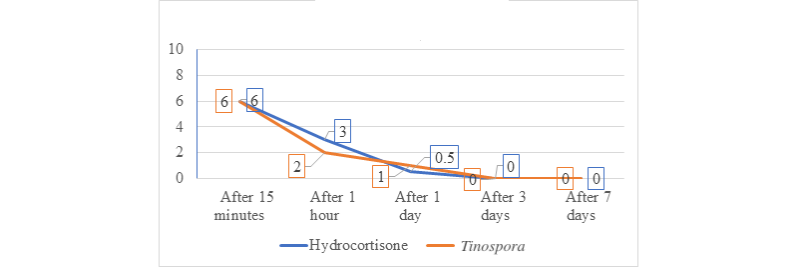
Graph showing the comparison of change in the median of all lesion size in mosquito bites treated with hydrocortisone 1% cream and *Tinospora* 25% cream during the 7-day study period.

The 29 participants who received hydrocortisone had a total of 126 lesions ranging from 1.0 to 16.0 mm, while 29 participants who received *Tinospora* had a total of 123 lesions ranging from 1.00 to 18.00 mm (Figures 7 and 8). Participants in both groups demonstrated a significant decrease in median lesion size from baseline to the seventh day (hydrocortisone: *P*<.001 and *Tinospora*: *P*<.001; Figure 9). Comparing the lesion sizes between the 2 groups, there were significant differences noted in the first hour (*P*=.003) and after 24 hours (*P*=.03). There were no significant differences between the 2 groups after 15 minutes and on the third and seventh day post treatment (Table 5).

**Table 5:** Median of all lesion sizes of participants with mosquito bites treated with hydrocortisone and *Tinospora rumphii.*.

			Hydrocortisone (mm), median (range)		*Tinospora* (mm), median (range)		*P* value^a^
**Time period**							
	After 15 minutes	7 (2-10)		7 (0-10)		.91	
	After 1 hour	1 (0-3)		0 (0-2)		.001	
	After 1 day	0 (0-3)		0 (0-0)		.005	
	After 3 days	0 (0-0)		0 (0-0)		N/A^b^	
	After 7 days	0 (0-0)		0 (0-0)		N/A	
*P* value^c^			.001		.001		N/A

^a^Significance set at *P*<.05 using the Wilcoxon rank sum test.

^b^N/A: not applicable.

^c^Significance set at *P*<.05 using the Friedman test.

### Visual Analog Score

**Figure 10:**
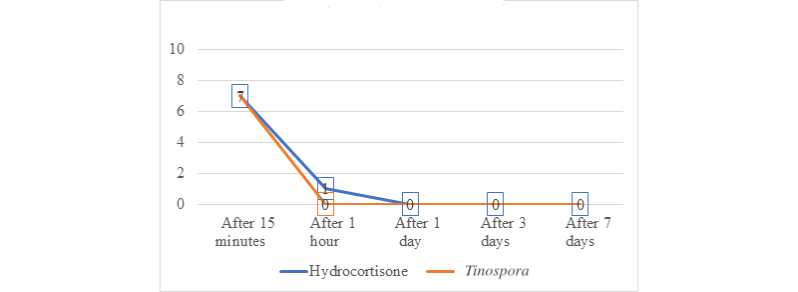
Comparison of changes in the median visual analog scores in mosquito bites treated with hydrocortisone 1% cream and *Tinospora* 25% cream during the 7-day study period.

Visual analog scores for both groups significantly decreased over time (hydrocortisone: *P*<.001 and *Tinospora*: *P*<.001; Figure 10). Baseline visual analog score for participants given hydrocortisone (median 7, range 2-10 mm) and *Tinospora* cream (median 7, range 0-10 mm) was not statistically different (*P*=.91). On days 3 and 7, there was absence of pruritus for both treatment groups. There was no statistically significant difference in the medians of visual analog scores for both treatment arms after 15 minutes. After 1 day and 3 days, a significant difference was observed (Table 6).

There were no reported adverse reactions using the 4-point scale for the 2 test products throughout the entire study period.

**Table 6:** Median visual analog score of participants with mosquito bites treated with hydrocortisone and Tinospora rumphii.

		Hydrocortisone (mm), median (range)	*Tinospora* (mm), median (range)	*P* value^a^
**Time period**				
	After 15 minutes	7 (2-10)	7 (0-10)	.91
	After 1 hour	1 (0-3)	0 (0-2)	.001
	After 1 day	0 (0-3)	0 (0-0)	.005
	After 3 days	0 (0-0)	0 (0-0)	N/A^b^
	After 7 days	0 (0-0)	0 (0-0)	N/A
*P* value^c^		.001	.001	N/A

^a^Significance set at *P*<.05 using the Wilcoxon rank sum test.

^b^N/A: not applicable.

^c^Significance set at *P*<.05 using the Friedman test.

## Discussion

### Principal Findings

*Tinospora* 25% cream is safe, effective, and comparable to hydrocortisone 1% cream as an anti-inflammatory and antipruritic agent in the management of mosquito bite reactions based on a decrease in lesion size, the proportion of participants with complete resolution of wheals, and improvement in pruritus intensity score using VAS. The CONSORT (Consolidated Standards of Reporting Trials) checklist can be found in Multimedia Appendix 2.

Our results showed a significant decrease in lesion size over time in both treatment groups. Between the 2 groups, there was a statistically significant difference after the first hour and after 24 hours. The timing of application of the topical agents may be significant due to the natural inflammatory course of the host to the mosquito bites. The reactions to mosquito bites can occur immediately after 1 to 2 hours and may be delayed as the lesions gradually enlarge over 24-48 hours after the bite [3]. Immediate reactions are mediated by IgE antibodies that bind to receptors with high affinity on mast cells causing mast cell degranulation along with other polymorphonuclear cells. Late-phase allergic reactions are mediated by lymphocytes and other polymorphonuclear cells such as eosinophils and neutrophils. In a study by Badar et al [28], decreased histamine-induced bronchospasm and a reduced number of disrupted mast cells were observed in animal studies. In an in vitro study by Chi et al [29], ethanol extracts of *Tinospora* revealed inhibitory activities on cyclooxygenase-1, cyclooxygenase-2, 5-lipoxygenase, and Pa2 with the IC_50_ values of 63.5, 81.2, 92.1, and 30.5 µg/mL, respectively. Decrease in the levels of inducible nitric oxide synthase, cyclooxygenase-2, and intracellular adhesion molecule-1 is responsible for the reduction in the release of proinflammatory mediators such as tumor necrosis factor-α, interleukin-4, nitric oxide, and IgE [29].

The efficacy of *Tinospora* species in the management of mosquito bite reactions can be explained by the presence of anti-inflammatory components such as sterols, triterpenes, flavonoids, alkaloids, saponins, glycosides, and tannins. Phytochemical analysis performed by DOST-ITDI on the *Tinospora* extract used in this study demonstrated the presence of the aforementioned active components. The most common phytosterols present in numerous amounts in the extracts of *Tinospora* were β-sitosterol. These sterols seem to target specific T-helper lymphocytes and result in improved T-lymphocyte and natural killer cell activity.

Aside from the decrease in lesion size, there was also complete resolution of lesions in both groups by day 3. Using the Friedman and Wilcoxon rank sum tests, there was no statistically significant difference in the change in mean lesion size for both treatment arms across all observation periods. Furthermore, a decreasing trend in the visual analog scores of the participants was observed. These suggest that the *Tinospora* cream has comparable efficacy to hydrocortisone cream.

There were no reported adverse reactions using the 4-point scale for the 2 test products. However, caution should always be observed in the use of standard of care such as corticosteroids because of their known side effects. These include local reactions such as epidermal atrophy, changes in pigmentation, contact dermatitis, and acneiform eruptions, among others. Furthermore, tachyphylaxis and rebound effects are also major concerns. Previous studies have demonstrated the safety of *Tinospora* cream. There were no cutaneous reactions observed in patch testing done on the participants in the study of Galang et al [22]. No adverse events were noted up to concentrations of 90% [22].

It is important to note that although the baseline characteristics are comparable, participants in the *Tinospora* group are younger compared to the hydrocortisone cream (*P*=.007). An epidemiological study by Kar et al [30] was similar to our study in that there was no age preponderance in the mosquito bites.

### Limitations

Among well-studied variables that attract mosquitoes are sex, carbon dioxide, body odor, secretions, and blood type. A study by Kulthanan et al [31] concluded that females were frequently bitten by mosquitoes due to their use of floral fragrances from perfumes. On the other hand, Rebollar-Tellez [32] reported that men were readily bitten due to having larger bodies that generate larger relative heat or carbon dioxide output. A study by Raji et al [33] showed that the loss of ionotropic receptor 8a (Ir8a) in *Aedes* species reduced the mosquitos’ attraction to humans and their odor. Shirai et al [34] demonstrated in their study that blood group O participants attracted more *Aedes* species as compared to groups with other blood types (B, AB, and A). Participants with blood type O possess components used by mosquitoes for the production of eggs. Some of these variables could have played a role, but these are beyond the scope of this study. This study did not take into account the attraction of mosquitoes to inherent human characteristics such as the color of skin and blood type.

### Recommendations

Future studies on *Tinospora* cream may include a concentration-response study in order to determine the ideal, safe, and effective doses of the compound. A quantitative phytochemical analysis along with an exploration of other vehicles (gel, lotion, cream, and ointment) to deliver the maximal dose is also recommended. A long-term safety study may also be performed. Furthermore, studies on children and patients with other inflammatory conditions such as psoriasis, atopic dermatitis, and other eczemas may be explored.

## Appendix

Multimedia Appendix 1 Plant authentication by the Republic of the Philippines Department of Agriculture Bureau of Plant Industry Manila.

Multimedia Appendix 2 CONSORT-EHEALTH checklist (V 1.6.1).

## Abbreviations

CRF: case report form
DOST-ITDI: Department of Science and Technology—Industrial Technology Development Institute
IgE: immunoglobulin E
IRB: institutional review board
VAS: visual analog scale
