# Supplementary material for: Efficacy and Safety of Makabuhay (Tinospora rumphii) 25% Cream Versus Hydrocortisone 1% Cream in the Management of Mosquito Bite Reactions: Randomized Double-Blind Controlled Trial
Source: JMIR Dermatol. 2023 Nov 8;6:e50380. doi: 10.2196/50380 (PMC10666022; doi:10.2196/50380)
Supplement: Multimedia Appendix 1 [file derma_v6i1e50380_app1.pdf]

Republic of the Philippines
Department of Agriculture
BUREAU OF PLANT INDUSTRY
Manila

692 San Andres Street
Malate, Manila
Philippines e-mail:
buplant@yahoo.com

Tel. No. 525-79-09
525-29-87
525-78-57
Fax No. 521-76-50

PLT-ID-CRPSD-1451-19
23 July 2019

PLANT IDENTIFICATION/CERTIFICATION

1. Local/Common Name:	Makabuhai (Tag.)
2. Family / Scientific Name:	Menispermaceae/*Tinospora rhumpii* Boerl.
3. Collector's Name & Address:	Rita C. Noble, MD and Julius G. Gatmaitan MD Research Institute for Tropical Medicine, Muntinlupa City
4. Collection Site/Source:	Silang, Cavite
5. Date of Collection:	19 July 2019
6. Types of Sample:	( ) Whole plant (x) Leaves<br>(x) Stem ( ) Roots<br>( ) Flowers ( ) Fruits<br>( ) Seeds ( ) others:
7. Status of Sample:	Fresh
8. Description/Remarks:	See attached sheet
9. Reference:	See attached sheet

Identified and Certified by:

MANUEL D. CHING
Chief, CIPGR Section

Noted by:

MARY ANN B. GUERRERO
Chief, CRPSD

HALAMAN AY YAMAN

**Family**	:	Menispermaceae
**Common Name**	:	Makabuhai (Tag.)
**Scientific Name**	:	*Tinospora rhumpii* Boerl.

DESCRIPTION/REMARKS

Makabuhai is a climbing dioecious vine reaching a height of 4 to 10 meters. The stem are up to 1 cm thick and somewhat fleshy, with scattered protuberances. The leaves are thin, ovate 6-12 cm in length and 7-12 cm in width with pointed and truncate somewhat cordate base and cuspidate to caudate tip, smooth and shining. The petioles are 3.5 to 6 cm long. The racemes are solitary or in pairs arising from the axils of fallen leaves, pale green slender 10 to 20 cm long. The flowers are pale green and long- pedicled. The fruit is 8 mm long.

The samples submitted are within the general characteristics of makabuhai. They differ only in sizes of the leaves and stem may be due diversity in location where they are grown.

Uses:

The vine is a universal medicine among the Filipinos and Malays. The aqueous extract is given for the treatment of stomach trouble, indigestion and diarrhea. It is also an effective remedy in the treatment of tropical ulcers. A preparation with coconut oil is an effective cure for rheumatism and for flatulence of children (kabag). The decoction of the stem is an excellent vulnerary for itches and cancerous wounds. Internally it is used as antimalaria and externally as parasiticides.

Reference:

QUISUMBING, E. 1978. Medicinal Plants of the Philippines. Katha Publishing Co. Inc. pp. 300-301.
