# Supplementary material for: Efficacy and Safety of Makabuhay (Tinospora rumphii) 25% Cream Versus Hydrocortisone 1% Cream in the Management of Mosquito Bite Reactions: Randomized Double-Blind Controlled Trial
Source: JMIR Dermatol. 2023 Nov 8;6:e50380. doi: 10.2196/50380 (PMC10666022; doi:10.2196/50380)
Supplement: Multimedia Appendix 2 [file derma_v6i1e50380_app2.pdf]

CONSORT-EHEALTH (V 1.6.1) - Submission/Publication Form

The CONSORT-EHEALTH checklist is intended for authors of randomized trials evaluating web-based and Internet-based applications/interventions, including mobile interventions, electronic games (incl multiplayer games), social media, certain telehealth applications, and other interactive and/or networked electronic applications. Some of the items (e.g. all subitems under item 5 - description of the intervention) may also be applicable for other study designs.

The goal of the CONSORT EHEALTH checklist and guideline is to be
a) a guide for reporting for authors of RCTs,
b) to form a basis for appraisal of an ehealth trial (in terms of validity)

CONSORT-EHEALTH items/subitems are MANDATORY reporting items for studies published in the Journal of Medical Internet Research and other journals / scientific societies endorsing the checklist.

Items numbered 1., 2., 3., 4a., 4b etc are original CONSORT or CONSORT-NPT (non-pharmacologic treatment) items.
Items with Roman numerals (i., ii, iii, iv etc.) are CONSORT-EHEALTH extensions/clarifications.

As the CONSORT-EHEALTH checklist is still considered in a formative stage, we would ask that you also RATE ON A SCALE OF 1-5 how important/useful you feel each item is FOR THE PURPOSE OF THE CHECKLIST and reporting guideline (optional).

Mandatory reporting items are marked with a red *.
In the textboxes, either copy & paste the relevant sections from your manuscript into this form - please include any quotes from your manuscript in QUOTATION MARKS,
or answer directly by providing additional information not in the manuscript, or elaborating on why the item was not relevant for this study.

YOUR ANSWERS WILL BE PUBLISHED AS A SUPPLEMENTARY FILE TO YOUR PUBLICATION IN JMIR AND ARE CONSIDERED PART OF YOUR PUBLICATION (IF ACCEPTED).
Please fill in these questions diligently. Information will not be copyedited, so please use proper spelling and grammar, use correct capitalization, and avoid abbreviations.

DO NOT FORGET TO SAVE AS PDF _AND_ CLICK THE SUBMIT BUTTON SO YOUR ANSWERS ARE IN OUR DATABASE !!!

Citation Suggestion (if you append the pdf as Appendix we suggest to cite this paper in the caption):
Eysenbach G, CONSORT-EHEALTH Group
CONSORT-EHEALTH: Improving and Standardizing Evaluation Reports of Web-based and Mobile Health Interventions
J Med Internet Res 2011;13(4):e126
URL: http://www.jmir.org/2011/4/e126/
doi: 10.2196/jmir.1923
PMID: 22209829

juliusggatmaitan@gmail.com Switch account
Not shared

* Indicates required question

Your name *
First Last

JULIUS GATMAITAN

Primary Affiliation (short), City, Country *
University of Toronto, Toronto, Canada

GATMAITAN MEDICAL AND SKIN CENTER

Your e-mail address *
abc@gmail.com

juliusggatmaitan@gmail.com

Title of your manuscript *
Provide the (draft) title of your manuscript.

Efficacy and Safety of Makabuhay (Tinospora rumphii) 25% Cream Versus Hydrocortisone 1% Cream in the Management of Mosquito Bite Reactions: Randomized Double-Blind Controlled Trial

Name of your App/Software/Intervention *
If there is a short and a long/alternate name, write the short name first and add the long name in brackets.

Makabuhay (Tinospora rumphii) 25% Cream

Evaluated Version (if any)
e.g. "V1", "Release 2017-03-01", "Version 2.0.27913"

Your answer

Language(s) *
What language is the intervention/app in? If multiple languages are available, separate by comma (e.g. "English, French")

Filipino

URL of your Intervention Website or App
e.g. a direct link to the mobile app on app in appstore (itunes, Google Play), or URL of the website. If the intervention is a DVD or hardware, you can also link to an Amazon page.

Your answer

URL of an image/screenshot (optional)

Your answer

Accessibility *
Can an enduser access the intervention presently?

- ( ) access is free and open
- ( ) access only for special usergroups, not open
- ( ) access is open to everyone, but requires payment/subscription/in-app purchases
- (x) app/intervention no longer accessible
- ( ) Other:

Primary Medical Indication/Disease/Condition *
e.g. "Stress", "Diabetes", or define the target group in brackets after the condition, e.g. "Autism (Parents of children with)", "Alzheimers (Informal Caregivers of)"

Mosquito Bite Reactions

Primary Outcomes measured in trial *
comma-separated list of primary outcomes reported in the trial

to compare the efficacy and safety of T rumph

Secondary/other outcomes
Are there any other outcomes the intervention is expected to affect?

Specifically, we will explore the proportion of participants experiencing complete resolution of wheals after 1 hour and on days 1, 3, and 7 of the study period; the lesion size after 1 hour and on days 1, 3, and 7 of the study period; the pruritus intensity scores using a visual analog scale (VAS) after 1 hour and on days 1, 3, and 7 of the study period; and the proportion of participants experiencing adverse effects

Recommended "Dose" *
What do the instructions for users say on how often the app should be used?

- (x) Approximately Daily
- ( ) Approximately Weekly
- ( ) Approximately Monthly
- ( ) Approximately Yearly
- ( ) "as needed"
- ( ) Other:

Approx. Percentage of Users (starters) still using the app as recommended after 3 months *

- (x) unknown / not evaluated
- ( ) 0-10%
- ( ) 11-20%
- ( ) 21-30%
- ( ) 31-40%
- ( ) 41-50%
- ( ) 51-60%
- ( ) 61-70%
- ( ) 71%-80%
- ( ) 81-90%
- ( ) 91-100%
- ( ) Other:

Overall, was the app/intervention effective? *

- ( ) yes: all primary outcomes were significantly better in intervention group vs control
- ( ) partly: SOME primary outcomes were significantly better in intervention group vs control
- (x) no statistically significant difference between control and intervention
- ( ) potentially harmful: control was significantly better than intervention in one or more outcomes
- ( ) inconclusive: more research is needed
- ( ) Other:

Article Preparation Status/Stage *
At which stage in your article preparation are you currently (at the time you fill in this form)

- ( ) not submitted yet - in early draft status
- ( ) not submitted yet - in late draft status, just before submission
- ( ) submitted to a journal but not reviewed yet
- ( ) submitted to a journal and after receiving initial reviewer comments
- (x) submitted to a journal and accepted, but not published yet
- ( ) published
- ( ) Other:

Journal *
If you already know where you will submit this paper (or if it is already submitted), please provide the journal name (if it is not JMIR, provide the journal name under "other")

- ( ) not submitted yet / unclear where I will submit this
- (x) Journal of Medical Internet Research (JMIR)
- ( ) JMIR mHealth and UHealth
- ( ) JMIR Serious Games
- ( ) JMIR Mental Health
- ( ) JMIR Public Health
- ( ) JMIR Formative Research
- ( ) Other JMIR sister journal
- ( ) Other:

Is this a full powered effectiveness trial or a pilot/feasibility trial? *

- (x) Pilot/feasibility
- ( ) Fully powered

Manuscript tracking number *
If this is a JMIR submission, please provide the manuscript tracking number under "other" (The ms tracking number can be found in the submission acknowledgement email, or when you login as author in JMIR. If the paper is already published in JMIR, then the ms tracking number is the four-digit number at the end of the DOI, to be found at the bottom of each published article in JMIR)

- ( ) no ms number (yet) / not (yet) submitted to / published in JMIR
- (x) Other: 50380

TITLE AND ABSTRACT

1a) TITLE: Identification as a randomized trial in the title

1a) Does your paper address CONSORT item 1a? *
I.e does the title contain the phrase "Randomized Controlled Trial"? (if not, explain the reason under "other")

- (x) yes
- ( ) Other:

1a-i) Identify the mode of delivery in the title
Identify the mode of delivery. Preferably use "web-based" and/or "mobile" and/or "electronic game" in the title. Avoid ambiguous terms like "online", "virtual", "interactive". Use "Internet-based" only if Intervention includes non-web-based Internet components (e.g. email), use "computer-based" or "electronic" only if offline products are used. Use "virtual" only in the context of "virtual reality" (3-D worlds). Use "online" only in the context of "online support groups". Complement or substitute product names with broader terms for the class of products (such as "mobile" or "smart phone" instead of "iphone"), especially if the application runs on different platforms.

	1	2	3	4	5	
subitem not at all important	( )	( )	( )	( )	(x)	essential

Clear selection

Does your paper address subitem 1a-i? *
Copy and paste relevant sections from manuscript title (include quotes in quotation marks "like this" to indicate direct quotes from your manuscript), or elaborate on this item by providing additional information not in the ms, or briefly explain why the item is not applicable/relevant for your study

Efficacy and Safety of Makabuhay (Tinospora rumphii) 25% Cream Versus Hydrocortisone 1% Cream in the Management of Mosquito Bite Reactions: Randomized Double-Blind Controlled Trial

1a-ii) Non-web-based components or important co-interventions in title
Mention non-web-based components or important co-interventions in title, if any (e.g., "with telephone support").

	1	2	3	4	5	
subitem not at all important	( )	( )	( )	( )	(x)	essential

Clear selection

Does your paper address subitem 1a-ii?
Copy and paste relevant sections from manuscript title (include quotes in quotation marks "like this" to indicate direct quotes from your manuscript), or elaborate on this item by providing additional information not in the ms, or briefly explain why the item is not applicable/relevant for your study

Efficacy and Safety of Makabuhay (Tinospora rumphii) 25% Cream Versus Hydrocortisone 1% Cream in the Management of Mosquito Bite Reactions: Randomized Double-Blind Controlled Trial

1a-iii) Primary condition or target group in the title
Mention primary condition or target group in the title, if any (e.g., "for children with Type I Diabetes") Example: A Web-based and Mobile Intervention with Telephone Support for Children with Type I Diabetes: Randomized Controlled Trial

	1	2	3	4	5	
subitem not at all important	( )	( )	( )	( )	(x)	essential

Clear selection

Does your paper address subitem 1a-iii? *
Copy and paste relevant sections from manuscript title (include quotes in quotation marks "like this" to indicate direct quotes from your manuscript), or elaborate on this item by providing additional information not in the ms, or briefly explain why the item is not applicable/relevant for your study

Efficacy and Safety of Makabuhay (Tinospora rumphii) 25% Cream Versus Hydrocortisone 1% Cream in the Management of Mosquito Bite Reactions: Randomized Double-Blind Controlled Trial

1b) ABSTRACT: Structured summary of trial design, methods, results, and conclusions
NPT extension: Description of experimental treatment, comparator, care providers, centers, and blinding status.

1b-i) Key features/functionalities/components of the intervention and comparator in the METHODS section of the ABSTRACT
Mention key features/functionalities/components of the intervention and comparator in the abstract. If possible, also mention theories and principles used for designing the site. Keep in mind the needs of systematic reviewers and indexers by including important synonyms. (Note: Only report in the abstract what the main paper is reporting. If this information is missing from the main body of text, consider adding it)

	1	2	3	4	5	
subitem not at all important	( )	( )	( )	( )	(x)	essential

Clear selection

Does your paper address subitem 1b-i? *
Copy and paste relevant sections from the manuscript abstract (include quotes in quotation marks "like this" to indicate direct quotes from your manuscript), or elaborate on this item by providing additional information not in the ms, or briefly explain why the item is not applicable/relevant for your study

The aim of the study is to assess the efficacy and safety of T rumphii 25% cream versus hydrocortisone 1% cream in the management of local cutaneous reactions caused by mosquito bites.

1b-ii) Level of human involvement in the METHODS section of the ABSTRACT
Clarify the level of human involvement in the abstract, e.g., use phrases like "fully automated" vs. "therapist/nurse/care provider/physician-assisted" (mention number and expertise of providers involved, if any). (Note: Only report in the abstract what the main paper is reporting. If this information is missing from the main body of text, consider adding it)

	1	2	3	4	5	
subitem not at all important	( )	( )	( )	( )	(x)	essential

Clear selection

Does your paper address subitem 1b-ii?
Copy and paste relevant sections from the manuscript abstract (include quotes in quotation marks "like this" to indicate direct quotes from your manuscript), or elaborate on this item by providing additional information not in the ms, or briefly explain why the item is not applicable/relevant for your study

Of the 70 participants screened for this study, which was approved by an institutional review board (IRB 2019-07) at the Dermatology Outpatient Department of the Research Institute for Tropical Medicine, Alabang, Muntinlupa, Philippines, 58 participants in total met the inclusion criteria and were randomized to treatment (Tinospora: n=29) and active control (hydrocortisone: n=29) groups.

1b-iii) Open vs. closed, web-based (self-assessment) vs. face-to-face assessments in the METHODS section of the ABSTRACT
Mention how participants were recruited (online vs. offline), e.g., from an open access website or from a clinic or a closed online user group (closed usergroup trial), and clarify if this was a purely web-based trial, or there were face-to-face components (as part of the intervention or for assessment). Clearly say if outcomes were self-assessed through questionnaires (as common in web-based trials). Note: In traditional offline trials, an open trial (open-label trial) is a type of clinical trial in which both the researchers and participants know which treatment is being administered. To avoid confusion, use "blinded" or "unblinded" to indicated the level of blinding instead of "open", as "open" in web-based trials usually refers to "open access" (i.e. participants can self-enrol). (Note: Only report in the abstract what the main paper is reporting. If this information is missing from the main body of text, consider adding it)

	1	2	3	4	5	
subitem not at all important	( )	( )	(x)	( )	( )	essential

Clear selection

Does your paper address subitem 1b-iii?
Copy and paste relevant sections from the manuscript abstract (include quotes in quotation marks "like this" to indicate direct quotes from your manuscript), or elaborate on this item by providing additional information not in the ms, or briefly explain why the item is not applicable/relevant for your study

Of the 70 participants screened for this study, which was approved by an institutional review board (IRB 2019-07) at the Dermatology Outpatient Department of the Research Institute for Tropical Medicine, Alabang, Muntinlupa, Philippines, 58 participants in total met the inclusion criteria and were randomized to treatment (Tinospora: n=29) and active control (hydrocortisone: n=29) groups.

1b-iv) RESULTS section in abstract must contain use data
Report number of participants enrolled/assessed in each group, the use/uptake of the intervention (e.g., attrition/adherence metrics, use over time, number of logins etc.), in addition to primary/secondary outcomes. (Note: Only report in the abstract what the main paper is reporting. If this information is missing from the main body of text, consider adding it)

	1	2	3	4	5	
subitem not at all important	( )	( )	( )	( )	(x)	essential

Clear selection

Does your paper address subitem 1b-iv?
Copy and paste relevant sections from the manuscript abstract (include quotes in quotation marks "like this" to indicate direct quotes from your manuscript), or elaborate on this item by providing additional information not in the ms, or briefly explain why the item is not applicable/relevant for your study

In total, 58 participants were randomized to receive Tinospora cream (n=29) or hydrocortisone cream (n=29). All participants completed the follow-up. There was a significant decrease in lesion size in both groups from the first 15 minutes to day 7 (P<.001). Comparing the lesion size in both groups, there was a statistically significant decrease in lesion size in the first hour (P=.003) and after 24 hours (P=.03). On day 1, 10% (n=29) of participants in the hydrocortisone group and 7% (n=29) in the Tinospora group experienced complete resolution. On day 3, all participants experienced complete resolution. No adverse effects were documented.

1b-v) CONCLUSIONS/DISCUSSION in abstract for negative trials
Conclusions/Discussions in abstract for negative trials: Discuss the primary outcome - if the trial is negative (primary outcome not changed), and the intervention was not used, discuss whether negative results are attributable to lack of uptake and discuss reasons. (Note: Only report in the abstract what the main paper is reporting. If this information is missing from the main body of text, consider adding it)

	1	2	3	4	5	
subitem not at all important	( )	( )	( )	( )	(x)	essential

Clear selection

Does your paper address subitem 1b-v?
Copy and paste relevant sections from the manuscript abstract (include quotes in quotation marks "like this" to indicate direct quotes from your manuscript), or elaborate on this item by providing additional information not in the ms, or briefly explain why the item is not applicable/relevant for your study

Tinospora 25% cream is safe, effective, and comparable to hydrocortisone 1% cream as an anti-inflammatory agent for mosquito bite reactions based on the decrease in lesion size, the proportion of participants with complete resolution of wheals, and improvement in pruritus intensity score using a visual analog scale. Long-term safety studies are recommended.

INTRODUCTION

2a) In INTRODUCTION: Scientific background and explanation of rationale

2a-i) Problem and the type of system/solution
Describe the problem and the type of system/solution that is object of the study: intended as stand-alone intervention vs. incorporated in broader health care program? Intended for a particular patient population? Goals of the intervention, e.g., being more cost-effective to other interventions, replace or complement other solutions? (Note: Details about the intervention are provided in "Methods" under 5)

	1	2	3	4	5	
subitem not at all important	( )	( )	( )	( )	(x)	essential

Clear selection

Does your paper address subitem 2a-i? *
Copy and paste relevant sections from the manuscript (include quotes in quotation marks "like this" to indicate direct quotes from your manuscript), or elaborate on this item by providing additional information not in the ms, or briefly explain why the item is not applicable/relevant for your study

To date, there are no published clinical trials investigating the use of Tinospora species on cutaneous reactions due to mosquito bites. Steroids have been previously used for mosquito bite reactions. Its misuse may be associated with adverse effects such as pigmentary changes, striae, skin atrophy, glaucoma (if used around the eyes), acneiform eruptions, and bruising, among others. The anti-inflammatory potential of T rumphii may provide a safe and natural alternative therapy for mosquito bite reactions.

2a-ii) Scientific background, rationale: What is known about the (type of) system
Scientific background, rationale: What is known about the (type of) system that is the object of the study (be sure to discuss the use of similar systems for other conditions/diagnoses, if appropiate), motivation for the study, i.e. what are the reasons for and what is the context for this specific study, from which stakeholder viewpoint is the study performed, potential impact of findings [2]. Briefly justify the choice of the comparator.

	1	2	3	4	5	
subitem not at all important	( )	( )	( )	( )	(x)	essential

Clear selection

Does your paper address subitem 2a-ii? *
Copy and paste relevant sections from the manuscript (include quotes in quotation marks "like this" to indicate direct quotes from your manuscript), or elaborate on this item by providing additional information not in the ms, or briefly explain why the item is not applicable/relevant for your study

Tinospora rumphii, synonymous with Tinospora cordifolia and Tinospora crispa and locally known as Makabuhay, is a large, glabrous, deciduous climbing shrub belonging to the family Menispermaceae, distributed in Southeast Asia [14]. In vitro and in vivo studies have demonstrated immunomodulatory [15], antiangiogenic [16], anti-inflammatory [17], and antipruritic [18] properties of Tinospora species. These properties have been postulated to be due to the alkaloids, diterpenoid lactones, glycosides, steroids, sesquiterpenoid, phenolics, aliphatic compounds, and polysaccharides present in the Tcordifolia plant

2b) In INTRODUCTION: Specific objectives or hypotheses

Does your paper address CONSORT subitem 2b? *
Copy and paste relevant sections from the manuscript (include quotes in quotation marks "like this" to indicate direct quotes from your manuscript), or elaborate on this item by providing additional information not in the ms, or briefly explain why the item is not applicable/relevant for your study

Specifically, we will explore the proportion of participants experiencing complete resolution of wheals after 1 hour and on days 1, 3, and 7 of the study period; the lesion size after 1 hour and on days 1, 3, and 7 of the study period; the pruritus intensity scores using a visual analog scale (VAS) after 1 hour and on days 1, 3, and 7 of the study period; and the proportion of participants experiencing adverse effects.

METHODS

[illegible]

3a) Description of trial design (such as parallel, factorial) including allocation ratio

Does your paper address CONSORT subitem 3a? *

Copy and paste relevant sections from the manuscript (include quotes in quotation marks "like this" to indicate direct quotes from your manuscript), or elaborate on this item by providing additional information not in the ms, or briefly explain why the item is not applicable/relevant for your study

This was a parallel-group, double-blind, randomized, placebo-controlled trial with a 1-week duration. This study was conducted at the Dermatology Outpatient Department and the Entomology Laboratory of the Research Institute for Tropical Medicine, Alabang, Muntinlupa, Philippines. The study was conducted over a span of 3 months from June 2019 to August 2019.

3b) Important changes to methods after trial commencement (such as eligibility criteria), with reasons

Does your paper address CONSORT subitem 3b? *

Copy and paste relevant sections from the manuscript (include quotes in quotation marks "like this" to indicate direct quotes from your manuscript), or elaborate on this item by providing additional information not in the ms, or briefly explain why the item is not applicable/relevant for your study

The inclusion criteria are the following: healthy men or women aged between 18 and 60 years, participants willing to sign an informed consent form after having read and understood its content upon the investigator's explanation, participants willing to comply with the study protocol requirements, and participants willing to have photos taken for documentation purposes.

3b-i) Bug fixes, Downtimes, Content Changes

Bug fixes, Downtimes, Content Changes: ehealth systems are often dynamic systems. A description of changes to methods therefore also includes important changes made on the intervention or comparator during the trial (e.g., major bug fixes or changes in the functionality or content) (5-iii) and other "unexpected events" that may have influenced study design such as staff changes, system failures/downtimes, etc. [2].

	1	2	3	4	5	
subitem not at all important	◉	○	○	○	○	essential

Clear selection

Does your paper address subitem 3b-i?

Copy and paste relevant sections from the manuscript (include quotes in quotation marks "like this" to indicate direct quotes from your manuscript), or elaborate on this item by providing additional information not in the ms, or briefly explain why the item is not applicable/relevant for your study

N/A

4a) Eligibility criteria for participants

Does your paper address CONSORT subitem 4a? *

Copy and paste relevant sections from the manuscript (include quotes in quotation marks "like this" to indicate direct quotes from your manuscript), or elaborate on this item by providing additional information not in the ms, or briefly explain why the item is not applicable/relevant for your study

The inclusion criteria are the following: healthy men or women aged between 18 and 60 years, participants willing to sign an informed consent form after having read and understood its content upon the investigator's explanation, participants willing to comply with the study protocol requirements, and participants willing to have photos taken for documentation purposes. Exclusion criteria are the following: participants with a history of severe reactions to mosquito bites (swelling and induration several centimeters in diameter, extensive periorbital swelling, lip swelling, and extensive limb swelling), pregnant or lactating women, participants with severe physical or mental illness, participants with active skin lesions or other dermatological disorders (ie, psoriasis, atopic dermatitis, and other eczemas), and participants with prior or current use of any oral antihistamines or corticosteroids in the past 2 weeks.

4a-i) Computer / Internet literacy

Computer / Internet literacy is often an implicit "de facto" eligibility criterion - this should be explicitly clarified.

	1	2	3	4	5	
subitem not at all important	◉	○	○	○	○	essential

Clear selection

Does your paper address subitem 4a-i?

Copy and paste relevant sections from the manuscript (include quotes in quotation marks "like this" to indicate direct quotes from your manuscript), or elaborate on this item by providing additional information not in the ms, or briefly explain why the item is not applicable/relevant for your study

N/A

4a-ii) Open vs. closed, web-based vs. face-to-face assessments:

Open vs. closed, web-based vs. face-to-face assessments: Mention how participants were recruited (online vs. offline), e.g., from an open access website or from a clinic, and clarify if this was a purely web-based trial, or there were face-to-face components (as part of the intervention or for assessment), i.e., to what degree got the study team to know the participant. In online-only trials, clarify if participants were quasi-anonymous and whether having multiple identities was possible or whether technical or logistical measures (e.g., cookies, email confirmation, phone calls) were used to detect/prevent these.

	1	2	3	4	5	
subitem not at all important	○	○	○	○	◉	essential

Clear selection

Does your paper address subitem 4a-ii? *

Copy and paste relevant sections from the manuscript (include quotes in quotation marks "like this" to indicate direct quotes from your manuscript), or elaborate on this item by providing additional information not in the ms, or briefly explain why the item is not applicable/relevant for your study

Purposive sampling was used in this study. Participants were recruited through the following methods: (1) direct recruitment (talking with potential participants about the study and using considerable care that the person does not feel pressured to participate); (2) posting institutional review board (IRB)–approved advertisements on bulletin boards at the lobby, dermatology department, training center, and triage area; and (3) through referrals (physicians and colleagues).

4a-iii) Information giving during recruitment

Information given during recruitment. Specify how participants were briefed for recruitment and in the informed consent procedures (e.g., publish the informed consent documentation as appendix, see also item X26), as this information may have an effect on user self-selection, user expectation and may also bias results.

	1	2	3	4	5	
subitem not at all important	○	○	○	○	◉	essential

Clear selection

Does your paper address subitem 4a-iii?

Copy and paste relevant sections from the manuscript (include quotes in quotation marks "like this" to indicate direct quotes from your manuscript), or elaborate on this item by providing additional information not in the ms, or briefly explain why the item is not applicable/relevant for your study

Purposive sampling was used in this study. Participants were recruited through the following methods: (1) direct recruitment (talking with potential participants about the study and using considerable care that the person does not feel pressured to participate); (2) posting institutional review board (IRB)–approved advertisements on bulletin boards at the lobby, dermatology department, training center, and triage area; and (3) through referrals (physicians and colleagues).

4b) Settings and locations where the data were collected

Does your paper address CONSORT subitem 4b? *

Copy and paste relevant sections from the manuscript (include quotes in quotation marks "like this" to indicate direct quotes from your manuscript), or elaborate on this item by providing additional information not in the ms, or briefly explain why the item is not applicable/relevant for your study

Purposive sampling was used in this study. Participants were recruited through the following methods: (1) direct recruitment (talking with potential participants about the study and using considerable care that the person does not feel pressured to participate); (2) posting institutional review board (IRB)–approved advertisements on bulletin boards at the lobby, dermatology department, training center, and triage area; and (3) through referrals (physicians and colleagues).

4b-i) Report if outcomes were (self-)assessed through online questionnaires

Clearly report if outcomes were (self-)assessed through online questionnaires (as common in web-based trials) or otherwise.

	1	2	3	4	5	
subitem not at all important	○	○	◉	○	○	essential

Clear selection

Does your paper address subitem 4b-i? *

Copy and paste relevant sections from the manuscript (include quotes in quotation marks "like this" to indicate direct quotes from your manuscript), or elaborate on this item by providing additional information not in the ms, or briefly explain why the item is not applicable/relevant for your study

Purposive sampling was used in this study. Participants were recruited through the following methods: (1) direct recruitment (talking with potential participants about the study and using considerable care that the person does not feel pressured to participate); (2) posting institutional review board (IRB)–approved advertisements on bulletin boards at the lobby, dermatology department, training center, and triage area; and (3) through referrals (physicians and colleagues).

4b-ii) Report how institutional affiliations are displayed

Report how institutional affiliations are displayed to potential participants [on ehealth media], as affiliations with prestigious hospitals or universities may affect volunteer rates, use, and reactions with regards to an intervention.(Not a required item – describe only if this may bias results)

	1	2	3	4	5	
subitem not at all important	○	○	○	○	◉	essential

Clear selection

Does your paper address subitem 4b-ii?

Copy and paste relevant sections from the manuscript (include quotes in quotation marks "like this" to indicate direct quotes from your manuscript), or elaborate on this item by providing additional information not in the ms, or briefly explain why the item is not applicable/relevant for your study

Purposive sampling was used in this study. Participants were recruited through the following methods: (1) direct recruitment (talking with potential participants about the study and using considerable care that the person does not feel pressured to participate); (2) posting institutional review board (IRB)–approved advertisements on bulletin boards at the lobby, dermatology department, training center, and triage area; and (3) through referrals (physicians and colleagues).

5) The interventions for each group with sufficient details to allow replication, including how and when they were actually administered

5-i) Mention names, credential, affiliations of the developers, sponsors, and owners

Mention names, credential, affiliations of the developers, sponsors, and owners [6] (if authors/evaluators are owners or developer of the software, this needs to be declared in a "Conflict of interest" section or mentioned elsewhere in the manuscript).

	1	2	3	4	5	
subitem not at all important	◉	○	○	○	○	essential

Clear selection

Does your paper address subitem 5-i?

Copy and paste relevant sections from the manuscript (include quotes in quotation marks "like this" to indicate direct quotes from your manuscript), or elaborate on this item by providing additional information not in the ms, or briefly explain why the item is not applicable/relevant for your study

Our study is a pilot study on herbal medication and the authors had no conflict of interest

5-ii) Describe the history/development process

Describe the history/development process of the application and previous formative evaluations (e.g., focus groups, usability testing), as these will have an impact on adoption/use rates and help with interpreting results.

	1	2	3	4	5	
subitem not at all important	○	○	◉	○	○	essential

Clear selection

Does your paper address subitem 5-ii?

Copy and paste relevant sections from the manuscript (include quotes in quotation marks "like this" to indicate direct quotes from your manuscript), or elaborate on this item by providing additional information not in the ms, or briefly explain why the item is not applicable/relevant for your study

The fresh mature stems of T rumphii were collected from the Sierra Madre mountain range at Infanta, Quezon Province, Philippines. These were authenticated by a botanist at the Bureau of Plant Industry (Multimedia Appendix 1 [25]). The samples were brought to the Department of Science and Technology—Industrial Technology Development Institute (DOST-ITDI) for preparation of the extracts. The organic constituents from the air-dried and chopped T rumphii stems were obtained by soaking the stems in 95% ethanol for 48 hours. The soaked solution was passed through Whatman No. 1 filter paper, and the filtrate was concentrated in a vacuum rotary evaporator at 60 °C in order to reduce the volume. The crude extracts yielded were submitted to DOST-ITDI for phytochemical analysis. A licensed pharmacist formulated the Tinospora 25% cream. Cultures of the cream were performed to ensure the absence of contaminants. The test product used for the active control group was hydrocortisone 1% cream (Marife C Biscocho Pharmacy). Both creams were unscented and similar in consistency. These were placed in identical white jars. The jars were coded (1 or 2) by the pharmacist. The investigators were unaware of the contents of each container applied to the participants.

5-iii) Revisions and updating

Revisions and updating. Clearly mention the date and/or version number of the application/intervention (and comparator, if applicable) evaluated, or describe whether the intervention underwent major changes during the evaluation process, or whether the development and/or content was "frozen" during the trial. Describe dynamic components such as news feeds or changing content which may have an impact on the replicability of the intervention (for unexpected events see item 3b).

	1	2	3	4	5	
subitem not at all important	○	○	◉	○	○	essential

Clear selection

Does your paper address subitem 5-iii?

Copy and paste relevant sections from the manuscript (include quotes in quotation marks "like this" to indicate direct quotes from your manuscript), or elaborate on this item by providing additional information not in the ms, or briefly explain why the item is not applicable/relevant for your study

The fresh mature stems of T rumphii were collected from the Sierra Madre mountain range at Infanta, Quezon Province, Philippines. These were authenticated by a botanist at the Bureau of Plant Industry (Multimedia Appendix 1 [25]). The samples were brought to the Department of Science and Technology—Industrial Technology Development Institute (DOST-ITDI) for preparation of the extracts. The organic constituents from the air-dried and chopped T rumphii stems were obtained by soaking the stems in 95% ethanol for 48 hours. The soaked solution was passed through Whatman No. 1 filter paper, and the filtrate was concentrated in a vacuum rotary evaporator at 60 °C in order to reduce the volume. The crude extracts yielded were submitted to DOST-ITDI for phytochemical analysis. A licensed pharmacist formulated the Tinospora 25% cream. Cultures of the cream were performed to ensure the absence of contaminants. The test product used for the active control group was hydrocortisone 1% cream (Marife C Biscocho Pharmacy). Both creams were unscented and similar in consistency. These were placed in identical white jars. The jars were coded (1 or 2) by the pharmacist. The investigators were unaware of the contents of each container applied to the participants.

5-iv) Quality assurance methods

Provide information on quality assurance methods to ensure accuracy and quality of information provided [1], if applicable.

	1	2	3	4	5	
subitem not at all important	○	○	○	○	◉	essential

Clear selection

Does your paper address subitem 5-iv?

Copy and paste relevant sections from the manuscript (include quotes in quotation marks "like this" to indicate direct quotes from your manuscript), or elaborate on this item by providing additional information not in the ms, or briefly explain why the item is not applicable/relevant for your study

The fresh mature stems of T rumphii were collected from the Sierra Madre mountain range at Infanta, Quezon Province, Philippines. These were authenticated by a botanist at the Bureau of Plant Industry (Multimedia Appendix 1 [25]). The samples were brought to the Department of Science and Technology—Industrial Technology Development Institute (DOST-ITDI) for preparation of the extracts. The organic constituents from the air-dried and chopped T rumphii stems were obtained by soaking the stems in 95% ethanol for 48 hours. The soaked solution was passed through Whatman No. 1 filter paper, and the filtrate was concentrated in a vacuum rotary evaporator at 60 °C in order to reduce the volume. The crude extracts yielded were submitted to DOST-ITDI for phytochemical analysis. A licensed pharmacist formulated the Tinospora 25% cream. Cultures of the cream were performed to ensure the absence of contaminants. The test product used for the active control group was hydrocortisone 1% cream (Marife C Biscocho Pharmacy). Both creams were unscented and similar in consistency. These were placed in identical white jars. The jars were coded (1 or 2) by the pharmacist. The investigators were unaware of the contents of each container applied to the participants.

5-v) Ensure replicability by publishing the source code, and/or providing screenshots/screen-capture video, and/or providing flowcharts of the algorithms used

Ensure replicability by publishing the source code, and/or providing screenshots/screen-capture video, and/or providing flowcharts of the algorithms used. Replicability (i.e., other researchers should in principle be able to replicate the study) is a hallmark of scientific reporting.

	1	2	3	4	5	
subitem not at all important	○	○	○	○	◉	essential

Clear selection

Does your paper address subitem 5-v?

Copy and paste relevant sections from the manuscript (include quotes in quotation marks "like this" to indicate direct quotes from your manuscript), or elaborate on this item by providing additional information not in the ms, or briefly explain why the item is not applicable/relevant for your study

These were all addressed in the Methodology section of the study

5-vi) Digital preservation

Digital preservation: Provide the URL of the application, but as the intervention is likely to change or disappear over the course of the years; also make sure the intervention is archived (Internet Archive, webcitation.org, and/or publishing the source code or screenshots/videos alongside the article). As pages behind login screens cannot be archived, consider creating demo pages which are accessible without login.

	1	2	3	4	5	
subitem not at all important	○	○	○	○	◉	essential

Clear selection

Does your paper address subitem 5-vi?

Copy and paste relevant sections from the manuscript (include quotes in quotation marks "like this" to indicate direct quotes from your manuscript), or elaborate on this item by providing additional information not in the ms, or briefly explain why the item is not applicable/relevant for your study

These were all addressed in the Methodology section of the study

5-vii) Access

Access: Describe how participants accessed the application, in what setting/context, if they had to pay (or were paid) or not, whether they had to be a member of specific group. If known, describe how participants obtained "access to the platform and Internet" [1]. To ensure access for editors/reviewers/readers, consider to provide a "backdoor" login account or demo mode for reviewers/readers to explore the application (also important for archiving purposes, see vi).

	1	2	3	4	5	
subitem not at all important	◉	○	○	○	○	essential

Clear selection

Does your paper address subitem 5-vii? *

Copy and paste relevant sections from the manuscript (include quotes in quotation marks "like this" to indicate direct quotes from your manuscript), or elaborate on this item by providing additional information not in the ms, or briefly explain why the item is not applicable/relevant for your study

not applicable in our study

5-viii) Mode of delivery, features/functionalities/components of the intervention and comparator, and the theoretical framework

Describe mode of delivery, features/functionalities/components of the intervention and comparator, and the theoretical framework [6] used to design them (instructional strategy [1], behaviour change techniques, persuasive features, etc., see e.g., [7, 8] for terminology). This includes an in-depth description of the content (including where it is coming from and who developed it) [1]," whether [and how] it is tailored to individual circumstances and allows users to track their progress and receive feedback" [6]. This also includes a description of communication delivery channels and – if computer-mediated communication is a component – whether communication was synchronous or asynchronous [6]. It also includes information on presentation strategies [1], including page design principles, average amount of text on pages, presence of hyperlinks to other resources, etc. [1].

	1	2	3	4	5	
subitem not at all important	○	○	○	○	◉	essential

Clear selection

Does your paper address subitem 5-viii? *

Copy and paste relevant sections from the manuscript (include quotes in quotation marks "like this" to indicate direct quotes from your manuscript), or elaborate on this item by providing additional information not in the ms, or briefly explain why the item is not applicable/relevant for your study

These were all addressed in the Methodology section of the study

5-ix) Describe use parameters

Describe use parameters (e.g., intended "doses" and optimal timing for use). Clarify what instructions or recommendations were given to the user, e.g., regarding timing, frequency, heaviness of use, if any, or was the intervention used ad libitum.

	1	2	3	4	5	
subitem not at all important	○	○	○	○	◉	essential

Clear selection

Does your paper address subitem 5-ix?

Copy and paste relevant sections from the manuscript (include quotes in quotation marks "like this" to indicate direct quotes from your manuscript), or elaborate on this item by providing additional information not in the ms, or briefly explain why the item is not applicable/relevant for your study

During the whole test period, the participants were instructed not to rub or scratch their arms with mosquito bites. Any adverse cutaneous reaction such as burning and popular or vesicular or bullous eruptions was recorded. Each participant was given 1 jar of Tinospora 25% cream or hydrocortisone 1% cream (Figure 4). The first application of the cream was 15 minutes after the mosquito bites. The participants were advised to apply the cream twice a day thereafter on the lesions. To ensure compliance, they were provided a diary to record their application and were required to bring the test product container on every visit. The diary also contained an adverse reaction section to record any reactions. They were advised not to apply any other medication or emollient or take any oral antihistamines or corticosteroids during the study period. The same mild soap (Dove; Unilever) was given to all participants. Photos were taken after the exposure to the mosquitoes in the cage at 15 minutes (when there are mosquito bites), after 1 hour, and on days 1, 3, and 7 of the study period.

5-x) Clarify the level of human involvement

Clarify the level of human involvement (care providers or health professionals, also technical assistance) in the e-intervention or as co-intervention (detail number and expertise of professionals involved, if any, as well as "type of assistance offered, the timing and frequency of the support, how it is initiated, and the medium by which the assistance is delivered". It may be necessary to distinguish between the level of human involvement required for the trial, and the level of human involvement required for a routine application outside of a RCT setting (discuss under item 21 – generalizability).

	1	2	3	4	5	
subitem not at all important	○	○	○	○	◉	essential

Clear selection

Does your paper address subitem 5-x?

Copy and paste relevant sections from the manuscript (include quotes in quotation marks "like this" to indicate direct quotes from your manuscript), or elaborate on this item by providing additional information not in the ms, or briefly explain why the item is not applicable/relevant for your study

To ensure blinding of the outcome assessor, a research assistant was hired for the collation of data. A standardized CRF approved by the IRB was used. CRFs were checked for completeness and errors by the primary investigators. Data were encoded by the research assistant in Microsoft Excel 2017 (Microsoft Corp). Collated data were given to the statistician for data analysis. All statistical analyses were performed using the R statistical package (version 3.2.0; R Foundation for Statistical Computing). Data on age were summarized using means and SDs. Frequencies and proportions were shown to describe sex and complete resolution of lesions. The size of lesions and pruritus scores were summarized using median and IQR. The t test for independent means was used to determine significant differences between the 2 groups in terms of age. It was a 2-tailed test. A chi-square test was conducted to determine if there were significant differences between the proportion of participants with complete resolution of mosquito bites (primary outcome measure) and the proportion of patients experiencing adverse effects. This test was also used to compare the age distribution of the 2 groups. The median of all lesion sizes and the median visual analog scores between hydrocortisone and Tinospora were compared using the Wilcoxon rank sum test. Friedman test was used to compare the change in lesion sizes and visual analog scores across a time period between hydrocortisone and Tinospora. No intention-to-treat analysis was done since there were no dropouts. Test results with P<.05 were considered to be statistically significant. The flow of participants through each stage of the trial is further seen in

5-xi) Report any prompts/reminders used

Report any prompts/reminders used: Clarify if there were prompts (letters, emails, phone calls, SMS) to use the application, what triggered them, frequency etc. It may be necessary to distinguish between the level of prompts/reminders required for the trial, and the level of prompts/reminders for a routine application outside of a RCT setting (discuss under item 21 – generalizability).

	1	2	3	4	5	
subitem not at all important	○	○	○	○	◉	essential

Clear selection

Does your paper address subitem 5-xi? *

Copy and paste relevant sections from the manuscript (include quotes in quotation marks "like this" to indicate direct quotes from your manuscript), or elaborate on this item by providing additional information not in the ms, or briefly explain why the item is not applicable/relevant for your study

This study was stopped in participants who experienced adverse reactions (severe pruritus, erythema, burning sensation, and vesicular or bullous eruption) to the creams or worsening of the mosquito bites (redness, warmth, swelling or induration that ranged from 2 to more than 10 cm in diameter, periorbital edema, extensive periorbital swelling, lip swelling, and extensive limb swelling). Participants were monitored based on World Health Organization guidelines for monitoring and reporting of adverse effects. Once a severe reaction occurred, the results were recorded, and participants were given the necessary medications. Unblinding was not necessary as the treatment would not have been different. Participants who did not comply with the protocol of applying test creams twice per day and those who applied other products or medications other than those stated in the protocol were considered to be withdrawn from the study. Dropouts referred to participants who were unable to return for assessment of lesions, and their outcomes were unknown by the end of the study period. The experiment was conducted in a controlled environment, with physicians trained in basic and advanced life support on standby. Although exceedingly rare, if participants experienced signs and symptoms of anaphylaxis, first aid was administered. Medications for anaphylaxis and other foreseeable adverse events, such as epinephrine, diphenhydramine, and intravenous and oral steroids, among others, were made accessible and available. Participants were brought to the nearby emergency room, which was within the vicinity of the outpatient clinic. Medical expenses for adverse events related to the study were shouldered by the study investigators. Participants could be withdrawn from the study due to (1) adverse events, (2) failure of therapy, (3) poor compliance, (4) lost to follow-up, and (5) voluntarily.

5-xii) Describe any co-interventions (incl. training/support)

Describe any co-interventions (incl. training/support): Clearly state any interventions that are provided in addition to the targeted eHealth intervention, as ehealth intervention may not be designed as stand-alone intervention. This includes training sessions and support [1]. It may be necessary to distinguish between the level of training required for the trial, and the level of training for a routine application outside of a RCT setting (discuss under item 21 – generalizability.

	1	2	3	4	5	
subitem not at all important	◉	○	○	○	○	essential

Clear selection

Does your paper address subitem 5-xii? *

Copy and paste relevant sections from the manuscript (include quotes in quotation marks "like this" to indicate direct quotes from your manuscript), or elaborate on this item by providing additional information not in the ms, or briefly explain why the item is not applicable/relevant for your study

not applicable in our study

6a) Completely defined pre-specified primary and secondary outcome measures, including how and when they were assessed

Does your paper address CONSORT subitem 6a? *

Copy and paste relevant sections from the manuscript (include quotes in quotation marks "like this" to indicate direct quotes from your manuscript), or elaborate on this item by providing additional information not in the ms, or briefly explain why the item is not applicable/relevant for your study

The general objective of our study is to compare the efficacy and safety of T rumphii 25% cream versus hydrocortisone 1% cream in the management of local cutaneous reactions caused by mosquito bites. Specifically, we will explore the proportion of participants experiencing complete resolution of wheals after 1 hour and on days 1, 3, and 7 of the study period; the lesion size after 1 hour and on days 1, 3, and 7 of the study period; the pruritus intensity scores using a visual analog scale (VAS) after 1 hour and on days 1, 3, and 7 of the study period; and the proportion of participants experiencing adverse effects.

6a-i) Online questionnaires: describe if they were validated for online use and apply CHERRIES items to describe how the questionnaires were designed/deployed

If outcomes were obtained through online questionnaires, describe if they were validated for online use and apply CHERRIES items to describe how the questionnaires were designed/deployed [9].

	1	2	3	4	5	
subitem not at all important	◉	○	○	○	○	essential

Clear selection

Does your paper address subitem 6a-i?

Copy and paste relevant sections from manuscript text

not applicable in our study

6a-ii) Describe whether and how “use” (including intensity of use/dosage) was defined/measured/monitored

Describe whether and how “use” (including intensity of use/dosage) was defined/measured/monitored (logins, logfile analysis, etc.). Use/adoption metrics are important process outcomes that should be reported in any ehealth trial.

	1	2	3	4	5	
subitem not at all important	○	○	○	○	◉	essential

Clear selection

Does your paper address subitem 6a-ii?

Copy and paste relevant sections from manuscript text

During the whole test period, the participants were instructed not to rub or scratch their arms with mosquito bites. Any adverse cutaneous reaction such as burning and popular or vesicular or bullous eruptions was recorded. Each participant was given 1 jar of Tinospora 25% cream or hydrocortisone 1% cream (Figure 4). The first application of the cream was 15 minutes after the mosquito bites. The participants were advised to apply the cream twice a day thereafter on the lesions. To ensure compliance, they were provided a diary to record their application and were required to bring the test product container on every visit. The diary also contained an adverse reaction section to record any reactions. They were advised not to apply any other medication or emollient or take any oral antihistamines or corticosteroids during the study period. The same mild soap (Dove; Unilever) was given to all participants. Photos were taken after the exposure to the mosquitoes in the cage at 15 minutes (when there are mosquito bites), after 1 hour, and on days 1, 3, and 7 of the study period.

6a-iii) Describe whether, how, and when qualitative feedback from participants was obtained

Describe whether, how, and when qualitative feedback from participants was obtained (e.g., through emails, feedback forms, interviews, focus groups).

	1	2	3	4	5	
subitem not at all important	○	○	○	○	◉	essential

Clear selection

Does your paper address subitem 6a-iii?

Copy and paste relevant sections from manuscript text

During the whole test period, the participants were instructed not to rub or scratch their arms with mosquito bites. Any adverse cutaneous reaction such as burning and popular or vesicular or bullous eruptions was recorded. Each participant was given 1 jar of Tinospora 25% cream or hydrocortisone 1% cream (Figure 4). The first application of the cream was 15 minutes after the mosquito bites. The participants were advised to apply the cream twice a day thereafter on the lesions. To ensure compliance, they were provided a diary to record their application and were required to bring the test product container on every visit. The diary also contained an adverse reaction section to record any reactions. They were advised not to apply any other medication or emollient or take any oral antihistamines or corticosteroids during the study period. The same mild soap (Dove; Unilever) was given to all participants. Photos were taken after the exposure to the mosquitoes in the cage at 15 minutes (when there are mosquito bites), after 1 hour, and on days 1, 3, and 7 of the study period.

6b) Any changes to trial outcomes after the trial commenced, with reasons

Does your paper address CONSORT subitem 6b? *

Copy and paste relevant sections from the manuscript (include quotes in quotation marks "like this" to indicate direct quotes from your manuscript), or elaborate on this item by providing additional information not in the ms, or briefly explain why the item is not applicable/relevant for your study

not applicable in our study

7a) How sample size was determined

NPT: When applicable, details of whether and how the clustering by care provides or centers was addressed

7a-i) Describe whether and how expected attrition was taken into account when calculating the sample size

Describe whether and how expected attrition was taken into account when calculating the sample size.

	1	2	3	4	5	
subitem not at all important	○	○	○	○	◉	essential

Clear selection

Does your paper address subitem 7a-i?

Copy and paste relevant sections from manuscript title (include quotes in quotation marks "like this" to indicate direct quotes from your manuscript), or elaborate on this item by providing additional information not in the ms, or briefly explain why the item is not applicable/relevant for your study

The sample size was calculated using the formula for a difference in proportions, using a power of 80% and a 5% level of significance. The proportion of success for the T cordifolia group and the hydrocortisone group was assumed to be 75% and 45%, respectively, based on the results of a previous study [23,24]. Calculations indicated that a total of 38 participants were needed (19 participants in each study arm). We recruited 58 participants to allow for a 20% dropout rate.
α=level of significance=0.05, $1-\beta$=power=0.80, $f(\alpha-\beta)=7.85$
$\pi1$=the anticipated proportion in those in the control group=45%
$\pi2$=the anticipated proportion in those in the treatment group=75%

7b) When applicable, explanation of any interim analyses and stopping guidelines

Does your paper address CONSORT subitem 7b? *

Copy and paste relevant sections from the manuscript (include quotes in quotation marks "like this" to indicate direct quotes from your manuscript), or elaborate on this item by providing additional information not in the ms, or briefly explain why the item is not applicable/relevant for your study

This study was stopped in participants who experienced adverse reactions (severe pruritus, erythema, burning sensation, and vesicular or bullous eruption) to the creams or worsening of the mosquito bites (redness, warmth, swelling or induration that ranged from 2 to more than 10 cm in diameter, periorbital edema, extensive periorbital swelling, lip swelling, and extensive limb swelling). Participants were monitored based on World Health Organization guidelines for monitoring and reporting of adverse effects. Once a severe reaction occurred, the results were recorded, and participants were given the necessary medications. Unblinding was not necessary as the treatment would not have been different. Participants who did not comply with the protocol of applying test creams twice per day and those who applied other products or medications other than those stated in the protocol were considered to be withdrawn from the study. Dropouts referred to participants who were unable to return for assessment of lesions, and their outcomes were unknown by the end of the study period. The experiment was conducted in a controlled environment, with physicians trained in basic and advanced life support on standby. Although exceedingly rare, if participants experienced signs and symptoms of anaphylaxis, first aid was administered. Medications for anaphylaxis and other foreseeable adverse events, such as epinephrine, diphenhydramine, and intravenous and oral steroids, among others, were made accessible and available. Participants were brought to the nearby emergency room, which was within the vicinity of the outpatient clinic. Medical expenses for adverse events related to the study were shouldered by the study investigators. Participants could be withdrawn from the study due to (1) adverse events, (2) failure of therapy, (3) poor compliance, (4) lost to follow-up, and (5) voluntarily.

8a) Method used to generate the random allocation sequence

NPT: When applicable, how care providers were allocated to each trial group

Does your paper address CONSORT subitem 8a? *

Copy and paste relevant sections from the manuscript (include quotes in quotation marks "like this" to indicate direct quotes from your manuscript), or elaborate on this item by providing additional information not in the ms, or briefly explain why the item is not applicable/relevant for your study

The study statistician generated a list of random numbers (simple randomization 1:1) using the computer-generated list of random numbers. A third party not directly involved in the study was assigned to the treatment arms as either 1 (hydrocortisone) or 2 (Tinospora species). Sealed opaque envelopes were used to allocate participants to either treatment (1 or 2). The envelope contained either 1 or 2, and assigned coinvestigators dispensed the jars accordingly. Only the pharmacist knew which code (1 or 2) corresponds to Tinospora cream or hydrocortisone cream. A diary card was given to participants and evaluated by investigators to ensure the compliance of participants. At the end of the study period, all case report forms (CRFs) were collected.

8b) Type of randomisation; details of any restriction (such as blocking and block size)

Does your paper address CONSORT subitem 8b? *

Copy and paste relevant sections from the manuscript (include quotes in quotation marks "like this" to indicate direct quotes from your manuscript), or elaborate on this item by providing additional information not in the ms, or briefly explain why the item is not applicable/relevant for your study

The study statistician generated a list of random numbers (simple randomization 1:1) using the computer-generated list of random numbers. A third party not directly involved in the study was assigned to the treatment arms as either 1 (hydrocortisone) or 2 (Tinospora species). Sealed opaque envelopes were used to allocate participants to either treatment (1 or 2). The envelope contained either 1 or 2, and assigned coinvestigators dispensed the jars accordingly. Only the pharmacist knew which code (1 or 2) corresponds to Tinospora cream or hydrocortisone cream. A diary card was given to participants and evaluated by investigators to ensure the compliance of participants. At the end of the study period, all case report forms (CRFs) were collected.

9) Mechanism used to implement the random allocation sequence (such as sequentially numbered containers), describing any steps taken to conceal the sequence until interventions were assigned

Does your paper address CONSORT subitem 9? *

Copy and paste relevant sections from the manuscript (include quotes in quotation marks "like this" to indicate direct quotes from your manuscript), or elaborate on this item by providing additional information not in the ms, or briefly explain why the item is not applicable/relevant for your study

The study statistician generated a list of random numbers (simple randomization 1:1) using the computer-generated list of random numbers. A third party not directly involved in the study was assigned to the treatment arms as either 1 (hydrocortisone) or 2 (Tinospora species). Sealed opaque envelopes were used to allocate participants to either treatment (1 or 2). The envelope contained either 1 or 2, and assigned coinvestigators dispensed the jars accordingly. Only the pharmacist knew which code (1 or 2) corresponds to Tinospora cream or hydrocortisone cream. A diary card was given to participants and evaluated by investigators to ensure the compliance of participants. At the end of the study period, all case report forms (CRFs) were collected.

10) Who generated the random allocation sequence, who enrolled participants, and who assigned participants to interventions

Does your paper address CONSORT subitem 10? *

Copy and paste relevant sections from the manuscript (include quotes in quotation marks "like this" to indicate direct quotes from your manuscript), or elaborate on this item by providing additional information not in the ms, or briefly explain why the item is not applicable/relevant for your study

The study statistician generated a list of random numbers (simple randomization 1:1) using the computer-generated list of random numbers. A third party not directly involved in the study was assigned to the treatment arms as either 1 (hydrocortisone) or 2 (Tinospora species). Sealed opaque envelopes were used to allocate participants to either treatment (1 or 2). The envelope contained either 1 or 2, and assigned coinvestigators dispensed the jars accordingly. Only the pharmacist knew which code (1 or 2) corresponds to Tinospora cream or hydrocortisone cream. A diary card was given to participants and evaluated by investigators to ensure the compliance of participants. At the end of the study period, all case report forms (CRFs) were collected.

11a) If done, who was blinded after assignment to interventions (for example, participants, care providers, those assessing outcomes) and how

NPT: Whether or not administering co-interventions were blinded to group assignment

11a-i) Specify who was blinded, and who wasn’t

Specify who was blinded, and who wasn’t. Usually, in web-based trials it is not possible to blind the participants [1, 3] (this should be clearly acknowledged), but it may be possible to blind outcome assessors, those doing data analysis or those administering co-interventions (if any).

	1	2	3	4	5	
subitem not at all important	○	○	○	○	◉	essential

Clear selection

Does your paper address subitem 11a-i? *

Copy and paste relevant sections from the manuscript (include quotes in quotation marks "like this" to indicate direct quotes from your manuscript), or elaborate on this item by providing additional information not in the ms, or briefly explain why the item is not applicable/relevant for your study

The study statistician generated a list of random numbers (simple randomization 1:1) using the computer-generated list of random numbers. A third party not directly involved in the study was assigned to the treatment arms as either 1 (hydrocortisone) or 2 (Tinospora species). Sealed opaque envelopes were used to allocate participants to either treatment (1 or 2). The envelope contained either 1 or 2, and assigned coinvestigators dispensed the jars accordingly. Only the pharmacist knew which code (1 or 2) corresponds to Tinospora cream or hydrocortisone cream. A diary card was given to participants and evaluated by investigators to ensure the compliance of participants. At the end of the study period, all case report forms (CRFs) were collected.

11a-ii) Discuss e.g., whether participants knew which intervention was the “intervention of interest” and which one was the “comparator”

Informed consent procedures (4a-ii) can create biases and certain expectations - discuss e.g., whether participants knew which intervention was the “intervention of interest” and which one was the “comparator”.

	1	2	3	4	5	
subitem not at all important	○	○	○	○	◉	essential

Clear selection

Does your paper address subitem 11a-ii?

Copy and paste relevant sections from the manuscript (include quotes in quotation marks "like this" to indicate direct quotes from your manuscript), or elaborate on this item by providing additional information not in the ms, or briefly explain why the item is not applicable/relevant for your study

The study statistician generated a list of random numbers (simple randomization 1:1) using the computer-generated list of random numbers. A third party not directly involved in the study was assigned to the treatment arms as either 1 (hydrocortisone) or 2 (Tinospora species). Sealed opaque envelopes were used to allocate participants to either treatment (1 or 2). The envelope contained either 1 or 2, and assigned coinvestigators dispensed the jars accordingly. Only the pharmacist knew which code (1 or 2) corresponds to Tinospora cream or hydrocortisone cream. A diary card was given to participants and evaluated by investigators to ensure the compliance of participants. At the end of the study period, all case report forms (CRFs) were collected.

11b) If relevant, description of the similarity of interventions

(this item is usually not relevant for ehealth trials as it refers to similarity of a placebo or sham intervention to a active medication/intervention)

Does your paper address CONSORT subitem 11b? *

Copy and paste relevant sections from the manuscript (include quotes in quotation marks "like this" to indicate direct quotes from your manuscript), or elaborate on this item by providing additional information not in the ms, or briefly explain why the item is not applicable/relevant for your study

The fresh mature stems of T rumphii were collected from the Sierra Madre mountain range at Infanta, Quezon Province, Philippines. These were authenticated by a botanist at the Bureau of Plant Industry (Multimedia Appendix 1 [25]). The samples were brought to the Department of Science and Technology—Industrial Technology Development Institute (DOST-ITDI) for preparation of the extracts. The organic constituents from the air-dried and chopped T rumphii stems were obtained by soaking the stems in 95% ethanol for 48 hours. The soaked solution was passed through Whatman No. 1 filter paper, and the filtrate was concentrated in a vacuum rotary evaporator at 60 °C in order to reduce the volume. The crude extracts yielded were submitted to DOST-ITDI for phytochemical analysis. A licensed pharmacist formulated the Tinospora 25% cream. Cultures of the cream were performed to ensure the absence of contaminants. The test product used for the active control group was hydrocortisone 1% cream (Marife C Biscocho Pharmacy). Both creams were unscented and similar in consistency. These were placed in identical white jars. The jars were coded (1 or 2) by the pharmacist. The investigators were unaware of the contents of each container applied to the participants.

12a) Statistical methods used to compare groups for primary and secondary outcomes

NPT: When applicable, details of whether and how the clustering by care providers or centers was addressed

Does your paper address CONSORT subitem 12a? *

Copy and paste relevant sections from the manuscript (include quotes in quotation marks "like this" to indicate direct quotes from your manuscript), or elaborate on this item by providing additional information not in the ms, or briefly explain why the item is not applicable/relevant for your study

To ensure blinding of the outcome assessor, a research assistant was hired for the collation of data. A standardized CRF approved by the IRB was used. CRFs were checked for completeness and errors by the primary investigators. Data were encoded by the research assistant in Microsoft Excel 2017 (Microsoft Corp). Collated data were given to the statistician for data analysis. All statistical analyses were performed using the R statistical package (version 3.2.0; R Foundation for Statistical Computing). Data on age were summarized using means and SDs. Frequencies and proportions were shown to describe sex and complete resolution of lesions. The size of lesions and pruritus scores were summarized using median and IQR. The t test for independent means was used to determine significant differences between the 2 groups in terms of age. It was a 2-tailed test. A chi-square test was conducted to determine if there were significant differences between the proportion of participants with complete resolution of mosquito bites (primary outcome measure) and the proportion of patients experiencing adverse effects. This test was also used to compare the age distribution of the 2 groups. The median of all lesion sizes and the median visual analog scores between hydrocortisone and Tinospora were compared using the Wilcoxon rank sum test. Friedman test was used to compare the change in lesion sizes and visual analog scores across a time period between hydrocortisone and Tinospora. No intention-to-treat analysis was done since there were no dropouts. Test results with P<.05 were considered to be statistically significant. The flow of participants through each stage of the trial is further seen in Figure 6 [26].

12a-i) Imputation techniques to deal with attrition / missing values

Imputation techniques to deal with attrition / missing values: Not all participants will use the intervention/comparator as intended and attrition is typically high in ehealth trials. Specify how participants who did not use the application or dropped out from the trial were treated in the statistical analysis (a complete case analysis is strongly discouraged, and simple imputation techniques such as LOCF may also be problematic [4]).

	1	2	3	4	5	
subitem not at all important	○	○	○	○	◉	essential

Clear selection

Does your paper address subitem 12a-i? *

Copy and paste relevant sections from the manuscript (include quotes in quotation marks "like this" to indicate direct quotes from your manuscript), or elaborate on this item by providing additional information not in the ms, or briefly explain why the item is not applicable/relevant for your study

not applicable in our paper

12b) Methods for additional analyses, such as subgroup analyses and adjusted analyses

Does your paper address CONSORT subitem 12b? *

Copy and paste relevant sections from the manuscript (include quotes in quotation marks "like this" to indicate direct quotes from your manuscript), or elaborate on this item by providing additional information not in the ms, or briefly explain why the item is not applicable/relevant for your study

Your answer

X26) REB/IRB Approval and Ethical Considerations [recommended as subheading under "Methods"] (not a CONSORT item)

X26-i) Comment on ethics committee approval

	1	2	3	4	5	
subitem not at all important	○	○	○	○	◉	essential

Clear selection

Does your paper address subitem X26-i?

Copy and paste relevant sections from the manuscript (include quotes in quotation marks "like this" to indicate direct quotes from your manuscript), or elaborate on this item by providing additional information not in the ms, or briefly explain why the item is not applicable/relevant for your study

This study was approved by the IRB of the Research Institute for Tropical Medicine (IRB 2019-07), registered at the Philippine Health Research Registry (PHRR230716-005932), and conducted in accordance with the tenets of the Declaration of Helsinki 2013 and National Ethical Guidelines for Health Research 2017.

x26-ii) Outline informed consent procedures

Outline informed consent procedures e.g., if consent was obtained offline or online (how? Checkbox, etc.?), and what information was provided (see 4a-ii). See [6] for some items to be included in informed consent documents.

	1	2	3	4	5	
subitem not at all important	○	○	○	○	◉	essential

Clear selection

Does your paper address subitem X26-ii?

Copy and paste relevant sections from the manuscript (include quotes in quotation marks "like this" to indicate direct quotes from your manuscript), or elaborate on this item by providing additional information not in the ms, or briefly explain why the item is not applicable/relevant for your study

Those who fulfilled the inclusion criteria were recruited. Participants were brought to a private room, where the study protocol was explained. Written informed consent was secured by the investigators and participants. The participants were requested to return on a separate day for the study.

X26-iii) Safety and security procedures

Safety and security procedures, incl. privacy considerations, and any steps taken to reduce the likelihood or detection of harm (e.g., education and training, availability of a hotline)

	1	2	3	4	5	
subitem not at all important	○	○	○	○	◉	essential

Clear selection

Does your paper address subitem X26-iii?

Copy and paste relevant sections from the manuscript (include quotes in quotation marks "like this" to indicate direct quotes from your manuscript), or elaborate on this item by providing additional information not in the ms, or briefly explain why the item is not applicable/relevant for your study

The experiment was conducted in a controlled environment, with physicians trained in basic and advanced life support on standby. Although exceedingly rare, if participants experienced signs and symptoms of anaphylaxis, first aid was administered. Medications for anaphylaxis and other foreseeable adverse events, such as epinephrine, diphenhydramine, and intravenous and oral steroids, among others, were made accessible and available. Participants were brought to the nearby emergency room, which was within the vicinity of the outpatient clinic. Medical expenses for adverse events related to the study were shouldered by the study investigators. Participants could be withdrawn from the study due to (1) adverse events, (2) failure of therapy, (3) poor compliance, (4) lost to follow-up, and (5) voluntarily.

RESULTS

13a) For each group, the numbers of participants who were randomly assigned, received intended treatment, and were analysed for the primary outcome

NPT: The number of care providers or centers performing the intervention in each group and the number of patients treated by each care provider in each center

Does your paper address CONSORT subitem 13a? *

Copy and paste relevant sections from the manuscript (include quotes in quotation marks "like this" to indicate direct quotes from your manuscript), or elaborate on this item by providing additional information not in the ms, or briefly explain why the item is not applicable/relevant for your study

Your answer

13b) For each group, losses and exclusions after randomisation, together with reasons

Does your paper address CONSORT subitem 13b? (NOTE: Preferably, this is shown in a CONSORT flow diagram) *

Copy and paste relevant sections from the manuscript (include quotes in quotation marks "like this" to indicate direct quotes from your manuscript), or elaborate on this item by providing additional information not in the ms, or briefly explain why the item is not applicable/relevant for your study

Your answer

13b-i) Attrition diagram

Strongly recommended: An attrition diagram (e.g., proportion of participants still logging in or using the intervention/comparator in each group plotted over time, similar to a survival curve) or other figures or tables demonstrating usage/dose/engagement.

	1	2	3	4	5	
subitem not at all important	○	○	○	○	◉	essential

Clear selection

Does your paper address subitem 13b-i?

Copy and paste relevant sections from the manuscript or cite the figure number if applicable (include quotes in quotation marks "like this" to indicate direct quotes from your manuscript), or elaborate on this item by providing additional information not in the ms, or briefly explain why the item is not applicable/relevant for your study

this can be seen in figure 6 of our paper

14a) Dates defining the periods of recruitment and follow-up

Does your paper address CONSORT subitem 14a? *

Copy and paste relevant sections from the manuscript (include quotes in quotation marks "like this" to indicate direct quotes from your manuscript), or elaborate on this item by providing additional information not in the ms, or briefly explain why the item is not applicable/relevant for your study

The study was conducted over a span of 3 months from June 2019 to August 2019.

14a-i) Indicate if critical “secular events” fell into the study period

Indicate if critical “secular events” fell into the study period, e.g., significant changes in Internet resources available or “changes in computer hardware or Internet delivery resources”

	1	2	3	4	5	
subitem not at all important	◉	○	○	○	○	essential

Clear selection

Does your paper address subitem 14a-i?

Copy and paste relevant sections from the manuscript (include quotes in quotation marks "like this" to indicate direct quotes from your manuscript), or elaborate on this item by providing additional information not in the ms, or briefly explain why the item is not applicable/relevant for your study

not applicable in our study

14b) Why the trial ended or was stopped (early)

Does your paper address CONSORT subitem 14b? *

Copy and paste relevant sections from the manuscript (include quotes in quotation marks "like this" to indicate direct quotes from your manuscript), or elaborate on this item by providing additional information not in the ms, or briefly explain why the item is not applicable/relevant for your study

not applicable in our study

15) A table showing baseline demographic and clinical characteristics for each group
NPT: When applicable, a description of care providers (case volume, qualification, expertise, etc.) and centers (volume) in each group

Does your paper address CONSORT subitem 15? *
Copy and paste relevant sections from the manuscript (include quotes in quotation marks "like this" to indicate direct quotes from your manuscript), or elaborate on this item by providing additional information not in the ms, or briefly explain why the item is not applicable/relevant for your study

Your answer

15-i) Report demographics associated with digital divide issues
In ehealth trials it is particularly important to report demographics associated with digital divide issues, such as age, education, gender, social-economic status, computer/Internet/ehealth literacy of the participants, if known.

	1	2	3	4	5	
subitem not at all important	◉	○	○	○	○	essential

Clear selection

Does your paper address subitem 15-i? *
Copy and paste relevant sections from the manuscript (include quotes in quotation marks "like this" to indicate direct quotes from your manuscript), or elaborate on this item by providing additional information not in the ms, or briefly explain why the item is not applicable/relevant for your study

not applicable in our study

16) For each group, number of participants (denominator) included in each analysis and whether the analysis was by original assigned groups

16-i) Report multiple "denominators" and provide definitions
Report multiple "denominators" and provide definitions: Report N's (and effect sizes) "across a range of study participation [and use] thresholds" [1], e.g., N exposed, N consented, N used more than x times, N used more than y weeks, N participants "used" the intervention/comparator at specific pre-defined time points of interest (in absolute and relative numbers per group). Always clearly define "use" of the intervention.

	1	2	3	4	5	
subitem not at all important	○	○	○	○	◉	essential

Clear selection

Does your paper address subitem 16-i? *
Copy and paste relevant sections from the manuscript (include quotes in quotation marks "like this" to indicate direct quotes from your manuscript), or elaborate on this item by providing additional information not in the ms, or briefly explain why the item is not applicable/relevant for your study

this can be seen in the methodology and results section of our study

16-ii) Primary analysis should be intent-to-treat
Primary analysis should be intent-to-treat, secondary analyses could include comparing only "users", with the appropriate caveats that this is no longer a randomized sample (see 18-i).

	1	2	3	4	5	
subitem not at all important	○	○	○	○	○	essential

Does your paper address subitem 16-ii?
Copy and paste relevant sections from the manuscript (include quotes in quotation marks "like this" to indicate direct quotes from your manuscript), or elaborate on this item by providing additional information not in the ms, or briefly explain why the item is not applicable/relevant for your study

Your answer

17a) For each primary and secondary outcome, results for each group, and the estimated effect size and its precision (such as 95% confidence interval)

Does your paper address CONSORT subitem 17a? *
Copy and paste relevant sections from the manuscript (include quotes in quotation marks "like this" to indicate direct quotes from your manuscript), or elaborate on this item by providing additional information not in the ms, or briefly explain why the item is not applicable/relevant for your study

Your answer

17a-i) Presentation of process outcomes such as metrics of use and intensity of use
In addition to primary/secondary (clinical) outcomes, the presentation of process outcomes such as metrics of use and intensity of use (dose, exposure) and their operational definitions is critical. This does not only refer to metrics of attrition (13-b) (often a binary variable), but also to more continuous exposure metrics such as "average session length". These must be accompanied by a technical description how a metric like a "session" is defined (e.g., timeout after idle time) [1] (report under item 6a).

	1	2	3	4	5	
subitem not at all important	○	○	○	○	○	essential

Does your paper address subitem 17a-i?
Copy and paste relevant sections from the manuscript (include quotes in quotation marks "like this" to indicate direct quotes from your manuscript), or elaborate on this item by providing additional information not in the ms, or briefly explain why the item is not applicable/relevant for your study

Your answer

17b) For binary outcomes, presentation of both absolute and relative effect sizes is recommended

Does your paper address CONSORT subitem 17b? *
Copy and paste relevant sections from the manuscript (include quotes in quotation marks "like this" to indicate direct quotes from your manuscript), or elaborate on this item by providing additional information not in the ms, or briefly explain why the item is not applicable/relevant for your study

Your answer

18) Results of any other analyses performed, including subgroup analyses and adjusted analyses, distinguishing pre-specified from exploratory

Does your paper address CONSORT subitem 18? *
Copy and paste relevant sections from the manuscript (include quotes in quotation marks "like this" to indicate direct quotes from your manuscript), or elaborate on this item by providing additional information not in the ms, or briefly explain why the item is not applicable/relevant for your study

Your answer

18-i) Subgroup analysis of comparing only users
A subgroup analysis of comparing only users is not uncommon in ehealth trials, but if done, it must be stressed that this is a self-selected sample and no longer an unbiased sample from a randomized trial (see 16-iii).

	1	2	3	4	5	
subitem not at all important	○	○	○	○	○	essential

Does your paper address subitem 18-i?
Copy and paste relevant sections from the manuscript (include quotes in quotation marks "like this" to indicate direct quotes from your manuscript), or elaborate on this item by providing additional information not in the ms, or briefly explain why the item is not applicable/relevant for your study

Your answer

19) All important harms or unintended effects in each group
(for specific guidance see CONSORT for harms)

Does your paper address CONSORT subitem 19? *
Copy and paste relevant sections from the manuscript (include quotes in quotation marks "like this" to indicate direct quotes from your manuscript), or elaborate on this item by providing additional information not in the ms, or briefly explain why the item is not applicable/relevant for your study

Your answer

19-i) Include privacy breaches, technical problems
Include privacy breaches, technical problems. This does not only include physical "harm" to participants, but also incidents such as perceived or real privacy breaches [1], technical problems, and other unexpected/unintended incidents. "Unintended effects" also includes unintended positive effects [2].

	1	2	3	4	5	
subitem not at all important	○	○	○	○	○	essential

Does your paper address subitem 19-i?
Copy and paste relevant sections from the manuscript (include quotes in quotation marks "like this" to indicate direct quotes from your manuscript), or elaborate on this item by providing additional information not in the ms, or briefly explain why the item is not applicable/relevant for your study

Your answer

19-ii) Include qualitative feedback from participants or observations from staff/researchers
Include qualitative feedback from participants or observations from staff/researchers, if available, on strengths and shortcomings of the application, especially if they point to unintended/unexpected effects or uses. This includes (if available) reasons for why people did or did not use the application as intended by the developers.

	1	2	3	4	5	
subitem not at all important	○	○	○	○	○	essential

Does your paper address subitem 19-ii?
Copy and paste relevant sections from the manuscript (include quotes in quotation marks "like this" to indicate direct quotes from your manuscript), or elaborate on this item by providing additional information not in the ms, or briefly explain why the item is not applicable/relevant for your study

Your answer

DISCUSSION

22) Interpretation consistent with results, balancing benefits and harms, and considering other relevant evidence
NPT: In addition, take into account the choice of the comparator, lack of or partial blinding, and unequal expertise of care providers or centers in each group

22-i) Restate study questions and summarize the answers suggested by the data, starting with primary outcomes and process outcomes (use)
Restate study questions and summarize the answers suggested by the data, starting with primary outcomes and process outcomes (use).

	1	2	3	4	5	
subitem not at all important	○	○	○	○	○	essential

Does your paper address subitem 22-i? *
Copy and paste relevant sections from the manuscript (include quotes in quotation marks "like this" to indicate direct quotes from your manuscript), or elaborate on this item by providing additional information not in the ms, or briefly explain why the item is not applicable/relevant for your study

Your answer

22-ii) Highlight unanswered new questions, suggest future research
Highlight unanswered new questions, suggest future research.

	1	2	3	4	5	
subitem not at all important	○	○	○	○	○	essential

Does your paper address subitem 22-ii?
Copy and paste relevant sections from the manuscript (include quotes in quotation marks "like this" to indicate direct quotes from your manuscript), or elaborate on this item by providing additional information not in the ms, or briefly explain why the item is not applicable/relevant for your study

Your answer

20) Trial limitations, addressing sources of potential bias, imprecision, and, if relevant, multiplicity of analyses

20-i) Typical limitations in ehealth trials
Typical limitations in ehealth trials: Participants in ehealth trials are rarely blinded. Ehealth trials often look at a multiplicity of outcomes, increasing risk for a Type I error. Discuss biases due to non-use of the intervention/usability issues, biases through informed consent procedures, unexpected events.

	1	2	3	4	5	
subitem not at all important	○	○	○	○	○	essential

Does your paper address subitem 20-i? *
Copy and paste relevant sections from the manuscript (include quotes in quotation marks "like this" to indicate direct quotes from your manuscript), or elaborate on this item by providing additional information not in the ms, or briefly explain why the item is not applicable/relevant for your study

Your answer

21) Generalisability (external validity, applicability) of the trial findings
NPT: External validity of the trial findings according to the intervention, comparators, patients, and care providers or centers involved in the trial

21-i) Generalizability to other populations
Generalizability to other populations: In particular, discuss generalizability to a general Internet population, outside of a RCT setting, and general patient population, including applicability of the study results for other organizations

	1	2	3	4	5	
subitem not at all important	○	○	○	○	○	essential

Does your paper address subitem 21-i?
Copy and paste relevant sections from the manuscript (include quotes in quotation marks "like this" to indicate direct quotes from your manuscript), or elaborate on this item by providing additional information not in the ms, or briefly explain why the item is not applicable/relevant for your study

Your answer

21-ii) Discuss if there were elements in the RCT that would be different in a routine application setting
Discuss if there were elements in the RCT that would be different in a routine application setting (e.g., prompts/reminders, more human involvement, training sessions or other co-interventions) and what impact the omission of these elements could have on use, adoption, or outcomes if the intervention is applied outside of a RCT setting.

	1	2	3	4	5	
subitem not at all important	○	○	○	○	○	essential

Does your paper address subitem 21-ii?
Copy and paste relevant sections from the manuscript (include quotes in quotation marks "like this" to indicate direct quotes from your manuscript), or elaborate on this item by providing additional information not in the ms, or briefly explain why the item is not applicable/relevant for your study

Your answer

OTHER INFORMATION

23) Registration number and name of trial registry

Does your paper address CONSORT subitem 23? *
Copy and paste relevant sections from the manuscript (include quotes in quotation marks "like this" to indicate direct quotes from your manuscript), or elaborate on this item by providing additional information not in the ms, or briefly explain why the item is not applicable/relevant for your study

Your answer

24) Where the full trial protocol can be accessed, if available

Does your paper address CONSORT subitem 24? *
Cite a Multimedia Appendix, other reference, or copy and paste relevant sections from the manuscript (include quotes in quotation marks "like this" to indicate direct quotes from your manuscript), or elaborate on this item by providing additional information not in the ms, or briefly explain why the item is not applicable/relevant for your study

Your answer

25) Sources of funding and other support (such as supply of drugs), role of funders

Does your paper address CONSORT subitem 25? *
Copy and paste relevant sections from the manuscript (include quotes in quotation marks "like this" to indicate direct quotes from your manuscript), or elaborate on this item by providing additional information not in the ms, or briefly explain why the item is not applicable/relevant for your study

Your answer

X27) Conflicts of Interest (not a CONSORT item)

X27-i) State the relation of the study team towards the system being evaluated
In addition to the usual declaration of interests (financial or otherwise), also state the relation of the study team towards the system being evaluated, i.e., state if the authors/evaluators are distinct from or identical with the developers/sponsors of the intervention.

	1	2	3	4	5	
subitem not at all important	○	○	○	○	○	essential

Does your paper address subitem X27-i?
Copy and paste relevant sections from the manuscript (include quotes in quotation marks "like this" to indicate direct quotes from your manuscript), or elaborate on this item by providing additional information not in the ms, or briefly explain why the item is not applicable/relevant for your study

Your answer

About the CONSORT EHEALTH checklist

As a result of using this checklist, did you make changes in your manuscript? *

○ yes, major changes
○ yes, minor changes
○ no

What were the most important changes you made as a result of using this checklist?

Your answer

How much time did you spend on going through the checklist INCLUDING making changes in your manuscript *

Your answer

As a result of using this checklist, do you think your manuscript has improved? *

○ yes
○ no
○ Other:

Would you like to become involved in the CONSORT EHEALTH group?
This would involve for example becoming involved in participating in a workshop and writing an "Explanation and Elaboration" document

○ yes
○ no
○ Other:

Any other comments or questions on CONSORT EHEALTH

Your answer

STOP - Save this form as PDF before you click submit
To generate a record that you filled in this form, we recommend to generate a PDF of this page (on a Mac, simply select "print" and then select "print as PDF") before you submit it.

When you submit your (revised) paper to JMIR, please upload the PDF as supplementary file.

Don't worry if some text in the textboxes is cut off, as we still have the complete information in our database. Thank you!

Final step: Click submit !
Click submit so we have your answers in our database!

Submit   Clear form

Never submit passwords through Google Forms.

This content is neither created nor endorsed by Google. Report Abuse - Terms of Service - Privacy Policy

Google Forms
